# miR-3189-targeted GLUT3 repression by HDAC2 knockdown inhibits glioblastoma tumorigenesis through regulating glucose metabolism and proliferation

**DOI:** 10.1186/s13046-022-02305-5

**Published:** 2022-03-08

**Authors:** Sungmin Kwak, Seung-Ho Park, Sung-Hak Kim, Gi-Jun Sung, Ji-Hye Song, Ji-Hoon Jeong, Hyunhee Kim, Chang Hoon Ha, Seong Who Kim, Kyung-Chul Choi

**Affiliations:** 1 Department of Biomedical Sciences, Asan Medical Center, University of Ulsan College of Medicine, Seoul, 05505 Republic of Korea; 2 Department of Animal Science, Chonnam National University, Gwangju, Republic of Korea; 3 Korea Food Research Institute, Wanju-gun, Republic of Korea; 4 Department of Convergence Medicine, Asan Medical Center, University of Ulsan College of Medicine, Seoul, 05505 Republic of Korea; 5 Departments of Biochemistry and Molecular Biology, Asan Medical Center, University of Ulsan College of Medicine, Seoul, 05505 Republic of Korea

**Keywords:** HDAC2, Glioblastoma, Glioma stem cells, miR-3189, GLUT3

## Abstract

**Background:**

Epigenetic regulations frequently appear in Glioblastoma (GBM) and are highly associated with metabolic alterations. Especially, Histone deacetylases (HDACs) correlates with the regulation of tumorigenesis and cell metabolism in GBM progression, and HDAC inhibitors report to have therapeutic efficacy in GBM and other neurological diseases; however, GBM prevention and therapy by HDAC inhibition lacks a mechanism in the focus of metabolic reprogramming.

**Methods:**

HDAC2 highly express in GBM and is analyzed in TCGA/GEPIA databases. Therefore, HDAC2 knockdown affects GBM cell death. Analysis of RNA sequencing and qRT-PCR reveals that miR-3189 increases and GLUT3 decreases by HDAC2 knockdown. GBM tumorigenesis also examines by using in vivo orthotopic xenograft tumor models. The metabolism change in HDAC2 knockdown GBM cells measures by glucose uptake, lactate production, and OCR/ECAR analysis, indicating that HDAC2 knockdown induces GBM cell death by inhibiting GLUT3.

**Results:**

Notably, GLUT3 was suppressed by increasing miR-3189, demonstrating that miR-3189-mediated GLUT3 inhibition shows an anti-tumorigenic effect and cell death by regulating glucose metabolism in HDAC2 knockdown GBM.

**Conclusions:**

Our findings will demonstrate the central role of HDAC2 in GBM tumorigenesis through the reprogramming of glucose metabolism by controlling miR-3189-inhibited GLUT3 expression, providing a potential new therapeutic strategy for GBM treatment.

**Supplementary Information:**

The online version contains supplementary material available at 10.1186/s13046-022-02305-5.

## Background

Glioblastoma (GBM) is the most common and lethal type of primary malignant brain tumor and accounts for 12–15% of all brain tumors [1]. Many tumor cells in GBM are aggressive and can resist well-known therapies due to intratumoral heterogeneity, leading to a high mortality rate of GBM patients. GBM also has several genetic variants that cause glioblastoma [2, 3], and genomic instability via mutagenesis and epigenetic modification occurs in different GBM subtypes with a frequency of 3 ~ 50% [4]. Therefore, gene expression biomarkers that contrast cell types in glioblastomas are important for prognosis and treatment [5]. GBM is known to contain cancer stem cells (CSCs) involved in tumorigenesis, which grow in a nutrient-depleted microenvironment [6]. GSCs are a subpopulation of tumor cells with tissue stem cell characteristics and display self-renewal and tumorigenic properties. GSCs are resistant to chemotherapy and radiotherapy and play an important role in tumor recurrence [7].

Histone acetyltransferases (HATs) and histone deacetylases (HDACs) are two families of enzymes that regulate histone acetylation. HDACs are grouped into four classes by function and DNA sequence similarity. Class I, II, and IV (HDAC1–11) enzymes have a zinc-binding active site and are inhibited by trichostatin A (TSA), and Class III (SIRT1–7) enzymes are known NAD^+^-dependent proteins, but not affected by TSA. HDAC play an essential role in the epigenetic regulation of gene expression through their effect on chromatin structure by removing the acetyl groups on a histone of the specific gene [8, 9]. Excessive histone deacetylation levels are associated with cancer pathology, as decreased histone acetylation can inhibit the expression of tumor regulatory genes [10]. In particular, disruption of HDAC activity is associated with the development of various human cancers and is involved in regulating tumor progression, the cell cycle, apoptosis, angiogenesis, and tumor invasion [11]. In a previous study report, we reported the increase the treatment efficiency of GBM of the combined treatment of melatonin and vorinostat [12]. The related results to GBM treatment by HDAC inhibitors were reported in several studies. However, it has not been clearly reported which HDACs are involved in GBM growth, and the inhibition mechanism of GBM growth by HDAC2 knockdown has not been reported. Therefore, we identified the regulation mechanism of the growth inhibition through cancer metabolism changes of GBM by HDAC2 knockdown in this study.

Glucose transport through cell membranes is an essential requirement for cell metabolism regulated by glucose transporters (GLUT). High glucose consumption in tumor cells is associated with abnormal GLUT family expression, which has been observed in colorectal cancer, brain cancer, and lung cancer [13]. Glucose is the brain’s primary energy source, and as neuronal cells cannot store glucose, intracellular glucose transport is essential [14]. In general, glucose uptake of all brain cells is known to be the result of glucose transporters (GLUT). Neurons in the human brain continuously require the delivery of glucose from the blood since the demand for the highest energy. Thus, glucose transporter 3 (GLUT3) is the main GLUT in neurons [15]. GLUT3 was found to be highly expressed in brain tumor patients, as well as having a high affinity for glucose, and is significantly correlated with the pathological grade of GBM [16, 17]. However, the association between HDAC2 and GLUT3 in GBM is not investigated; the role of HDAC2 in the molecular mechanisms that regulate cell death in GBM progression is not well understood. Thus, we analyzed miRNA expression to investigate the potential role of GLUT3 by HDAC2 inhibition. Some miRNAs act as oncogenes, while others have been shown to act as tumor suppressors [18, 19]. We identified miR-3189, as a novel miRNA that inhibits GLUT3 expression, and miR-3189 possesses tumor suppressor functionality via inhibition of GLUT3 which is important for the control of cancer cell metabolism in GBM. These findings support the critical role of cancer metabolism and its epigenetic regulators in GBM, suggesting that the development of specific HDAC2 and GLUT3 inhibitors as potential therapies may increase GBM patients’ survival.

## Methods

More information details in Additional file 2 (Supplementary Tables).

### Human GBM cells and GSCs

GBM Cell lines (A172, U87MG, T98G, LN18, LN229, U118, U343, and U373) were purchased from ATCC and maintained in high glucose DMEM media supplemented with 10% FBS and 1% Antibiotics. GSC Cells (GSC20, GSC23, GSC28, and GSC267) were provided by E.P. Sulman, M.D., Ph.D. (University of Texas M.D. Anderson Cancer Center). To produce *HDAC2* knockdown GBM/GSC stable cells, cell lines were infected with Lentivirus carrying pLKO/TetON shControl (DOX-inducible shcontrol) and *HDAC2* shRNA (DOX-inducible shHDAC2) plasmids, and stable cells were selected by puromycin (5 μg/ml) and treated with doxycycline (2.5 μg/ml) for *HDAC2* knockdown (Supplementary Table 1).

### Immunoblotting assay

Cell extracts were prepared from human glioblastoma cell lines using RIPA buffer (20 mM Tris HCl, 150 mM NaCl, 1% Triton X-100, 1.5 mM MgCl2, 1 mM Na vanadate, 10% glycerol, 1 mM EDTA, and proteinase inhibitor, pH 7.5). Cell extracts were collected in 1 ml Eppendorf tubes, and protein levels were determined by absorbance at 660 nm following incubation with the protein assay reagent (Thermo Fisher, MA, USA). 50 μg of protein lysate extract per sample were mixed by SDS sample buffer (250 nM Tris-HCl pH 6.8, 10% SDS, 30% Glycerol, 5% β-Mercaptoethanol, 0.02% bromophenol blue). Each sample was separated by electrophoresis in 5–15% acrylamide gels. After the transfer, membranes were blocked with skim milk. The membrane was then incubated with a primary antibody. Each primary antibody incubated overnight at 4 °C. After washing three times for 10 min in PBS, membranes were treated with the secondary antibody. Secondary antibody was incubated for 2 h at room temperature. After washing, HRP reactions were initiated by using ECL Solution (Advansta). Protein bands were visualized utilizing enhanced chemiluminescence (Amersham Biosciences, Piscataway, USA) according to the manufacturer’s instruction. Western blot data was performed three times independently, and one representative image is shown.

### Immunofluorescence (IF) analysis

Cells were grown in 60 mm dishes to 50% confluency. Cells were fixed in 4% paraformaldehyde in PBS (pH 7.4) and washed in PBS 3 times, then permeabilized in 0.2% Triton X-100 in PBS for 5–10 min on ice. Primary antibodies were diluted in PBS at a concentration of 1:50 and incubated for 18 h in a 4 °C environment. After washing the slide three times, the secondary antibody was added and incubated at room temperature for 3 h. After washing three times, the cells were incubated with DAPI - Mayer’s Hematoxylin (Abcam, Cambridge, USA) at room temperature for 10 min, and then mounted on a cover slip. Cells subjected to fluorescence immunostaining were identified through a ZOE fluorescence cell imager (BioRAD, California, USA).

### Immunohistochemistry analysis

Human Brain Cancer Glioblastoma (grade IV) and normal brain samples were purchased from US Biomax, Inc., and mouse brain tissues was prepared in the Laboratory of Animal Research at the Asan Medical Center. The tissues were sectioned 4 μm thick on paraffin-embedded slides. Tissue slides were incubated at 60 °C for 1 h and then deparaffinized with xylenes and rehydrated with 100, 95, 90, 85, 50, and 0% ethanol. The primary antibody was incubated in the tissues overnight at 4 °C. Antibodies used include HDAC2 (Santa cruz, 1:1000), Bax (Cell signaling, 1:1000), Apaf-1 (Cell signaling, 1:1000), and GLUT3 (Cell signaling, 1:1000). The IHC process was carried out using the PROCAM IHC kit (Abcam, MA USA). Digital images were obtained through (OLYMPUS-cellSens Standard). Quantitative analysis of the images was performed using Image J (NIH).

### Study ethics approval and animal studies

Mice were maintained in the Asan Medical Center (AMC) SPF facility of the University of Ulsan College of Medicine in accordance with the International Animal Care and Use Committee (IACUC) guidelines. All experimental methods abided by the Helsinki Declaration. For all experiments, we used male BALB/C^nu/nu^ mice (8–10 weeks old) and purchased from Central Lab Animal, Inc. To measure in vivo brain tumorigenesis by orthotopic xenograft mouse model, mice were anesthetized using Avertin. The mouse skulls were fixed using a stereotactic device, and a hole was made in the skulls using a drill (SAESHIN, Strong207A). U87MG cells transformed with shControl or shHDAC2 using pLKO TetON vector were used. 10 mice in each experimental group were injected with 5 × 10^5^ cells resuspended in 10 μl PBS using a microinjector. Mice injected with U87MG cells were observed for 3 days. 5 mice were randomly selected from each group and given doxycycline (10 mg/kg) in drinking water. Mice were maintained for 5 weeks and then sacrificed.

### Microarray and GSEA analysis

A172 cells were grown at 37 °C in a 5% CO_2_ environment. Cells were treated with doxycycline and harvested after 48 h incubation. Total RNA was extracted from A172 cells and treated according to the protocol of RNeasy Plus Mini kit (Qiagen). Trizol extraction of total RNA was performed according to the manufacturer’s instructions. cDNA was synthesized using the GeneChip WT (Whole Transcript) Amplification kit as described by the manufacturer. The sense cDNA was then fragmented and biotin-labeled with TdT (terminal deoxynucleotidyl transferase) using the GeneChip WT Terminal labeling kit. 5.5 μg of labeled DNA target was hybridized to the Affymetrix GeneChip Human 2.0 ST Array at 45 °C for 16 h. Hybridized arrays were scanned on a GCS3000 Scanner (Affymetrix). Raw data were extracted automatically via the Affymetrix data extraction protocol using the software provided by Affymetrix GeneChip® Command Console® Software (AGCC). After importing CEL files, the data were summarized and normalized with the robust multi-average (RMA) method implemented in Affymetrix® Expression Console™ Software (EC).

### Measurement of mitochondria oxidation/ extracellular acidification rate

OCR and ECAR were measured using an XF96 extracellular flux analyzer (Seahorse Bioscience, Agilent). GBM cells were seeded 4 × 10^4^ cells/well on the XF96 plate (Agilent, 101,085–004). Cells were washed with Seahorse media, including dedicated glucose, glutamate, and pyruvate, followed by fresh media change. Plates were incubated for 1 h at 37 °C in an incubator without CO_2_. Oligomycin (Sigma, 75,351), FCCP (Sigma, C2920), Antimycin A (Sigma, A8674), and Rotenone (Sigma, R8875) was injected into the cartridge plate and set in the device. The incubated plate was then set in the instrument and automatic injection and measurement were performed.

### Cell viability

Cells were plated in 96-well plates at a density of 5 × 10^3^ cells/well in DMEM with 10% FBS and 1% antibiotics. Cell viability was determined by the MTT (3-(4,5-dimethylthiazol-2-yl)-2,5-diphenyltertrazolium bromide) assay (Sigma-Aldrich). After incubation for 24 h, 20 μL of the MTT reagent (2 mg/mL) was added to each well and incubated for 90 min at 37 °C in a CO_2_ incubator. Cells were then refreshed with complete DMEM media (10% FBS, 1% Antibiotics). Absorption was measured at 570 nm with a micro plate reader (Model 550, BIO-RAD Laboratories, Hercules, CA, USA). All MTT assay results were presented as the means ±SD of three independent experiments.

### Quantitative RealTime- polymerase chain reaction analysis (qRT-PCR)

Total RNA was isolated using Total RNA extraction kit (iNtRON). 500 ng of RNA was reverse transcribed using a high efficiency PrimeScript™ Reverse Transcriptase (TAKAR2 # 2680A). In addition, real time PCR was performed using an iNtRON Thermo scientific PIKOREAL 96 Real time PCR instrument and using 2X PCR Master Mix (ElpisBIO EBT-1801). Primer sequences were designed using Primer Express 3.0.1 software and NCBI primer-Blast. Primer sequences are described in the Supplementary Table 2. Each value was normalized to the expression level of *Gapdh* mRNA, and the measurement was repeated in triplicate. Data were analyzed using PikoReal Software 2.2 normalized as a control of the contents corresponding to the experimental group. qPCR data were determined through the average of CT (cycle threshold) values ​​and repeated in triplicate.

### Flow cytometry analysis

To assess the extent of apoptosis after DNA damage, cells were stained with both Annexin V-FITC and propidium iodide according to the manufacturer’s protocol using the Dead Cell Apoptosis Kit with Annexin V Alexa Fluor® 488 & PI for Flow Cytometry (Invitrogen, V13241) for 15 min at room temperature. Cells were analyzed using a BD FACS Canto II cytometer (Becton Dickinson).

### In vivo PET-MRI imaging analysis

PET-MRI fused imaging was performed using a nanoScanPET/MRI system (1 T, Mediso, Hungary). Mice were kept warm, and 7.5 ± 1.0 MBq in 0.2 mL of FDG was administered intravenously via the tail vein to keep the mouse under anesthesia (1.5% isoflurane in 100% O_2_ gas). MR brain imaging obtained T1 weighted with Gradient-echo (GRE) 3D sequence (TR = 25 ms, TEeff = 3, FOV = 50 mm, matrix = 128 × 128) and T2 weighted with Fast Spin Echo (FSE) 3D Sequence (TR = 2400 ms, TE eff = 110, FOV = 50 mm, matrix = 256 × 256) images, which were acquired during the FDG uptake period. 20 min of static PET images were acquired in a 1–3 coincident in a single field of view with MRI range. Body temperature was maintained with heated air (37 °C) on the animal bed (Multicell, Mediso, Hungary). PET images were reconstructed by Tera-Tomo 3D, in full detector mode, with all the corrections on, high regularization and 8 iterations. Three-dimensional volume of interest (VOI) analysis of the reconstructed images was performed using the InterView Fusion software package (Mediso, Hungary) and applying standard uptake value (SUV) analysis. VOI were fixed with a diameter of 1.5 mm sphere and were drawn for the tumor and cerebellum site. The SUV of each VOI sites was calculated using the formula: SUVmean = tumor radioactivity in the tumor volume of interest with the unit of Bq/cc × body weight (g) divided by injected radioactivity.

### Glucose uptake and lactate production

To glucose uptake assay, GBM cells were plated in 6-well plates at a density of 7 × 10^4^ cells/well in DMEM medium with 10% FBS and 1% antibiotics. Dox-inducible GBM cells were treated with doxycycline. After 48 h, cells were washed twice in PBS and incubated in glucose-free media for 1 h. The medium was then removed, and cells were incubated 1 mM 2-DG diluted in PBS for 20 min. Subsequent glucose uptake analysis was performed using a 2-Deoxyglucose Uptake measurement kit (Cosmobio, CSR-OKP0PMG-K01TE), and the intracellular glucose level was measured using a microplate reader at 420 nm. To lactate production assay, GBM cells were plated in 96-well plates at a density of 5 × 10^3^ cells/well in DMEM medium with 10% FBS and 1% antibiotics. Dox-inducible GBM cells were treated with doxycycline. After 48 h, lactate production analysis was performed using a lactate fluorometric assay kit (Biovision #K607) according to the manufacturer’s instructions. The wavelength of excitation and emission were 535 and 590 nm, respectively, and were measured using a microplate reader.

### Colony formation assay

GBM cells were seeded in 6-well plates at 1 × 10^3^ cells/well and incubated with CO_2_ at 37 °C for 48 h. GBM cells transfected with siRNA were incubated for 14 days. Colonies were washed twice with PBS and fixed with distilled water containing 10% methanol and 2% formaldehyde. Colonies were stained with 0.5% crystal violet/20% methanol/PBS. Colony numbers measured using Oxford Optronix – GELCOUNT™.

### Luciferase reporter assay

GBM cells were seeded in 96-well plates at 5 × 10^3^ cells/well and incubated with CO_2_ at 37 °C for 24 h. GBM cells transfected with TransIT® -LT1 (Mirus) were incubated for 48 h. After transfection, the luciferase levels were measured using a 2030 Multilabel Reader VICTOR X3 instrument (Perkin Elmer). Dual**-**Luciferase Reporter Assay System was measured according to the manufacturer’s instructions (Promega, E1980). All reporter activities were normalized to *Renilla* luciferase activity and are presented as the mean (± standard deviation) of three independent experiments.

### Quantification and statistical analysis

In each animal experiment, multiple mice were analyzed as biological replicates. The examination of histological sections and other experiments was carried out by a trained researcher who was not blinded to the study. No statistical methods were used to predetermine sample sizes, which were based on work in similar published research. No data were excluded from these analyses. Error bars ± SEM represent values of * *p* < 0.05, ** *p* < 0.01, ***p < 0.001 unless stated otherwise. Statistical parameters and biological replicates can be found in the respective Figure legends. Significance was determined using a two-tailed unpaired Student’s t-test when comparing two groups, or one-way ANOVA test when comparing three groups, and repeated-measures ANOVA to compare three or more matched groups. Graphpad/Prism software was used to conduct the statistical data analysis.

### Data availability

All datasets generated in this work have been deposited to Gene Expression Omnibus (GEO) under accession number GSE158355, available at https://www.ncbi.nlm.nih.gov/geo/query/ acc.cgi?acc = GSE158355.

## Results

### HDAC2 knockdown induces cell death in GBM

**Fig. 1:**
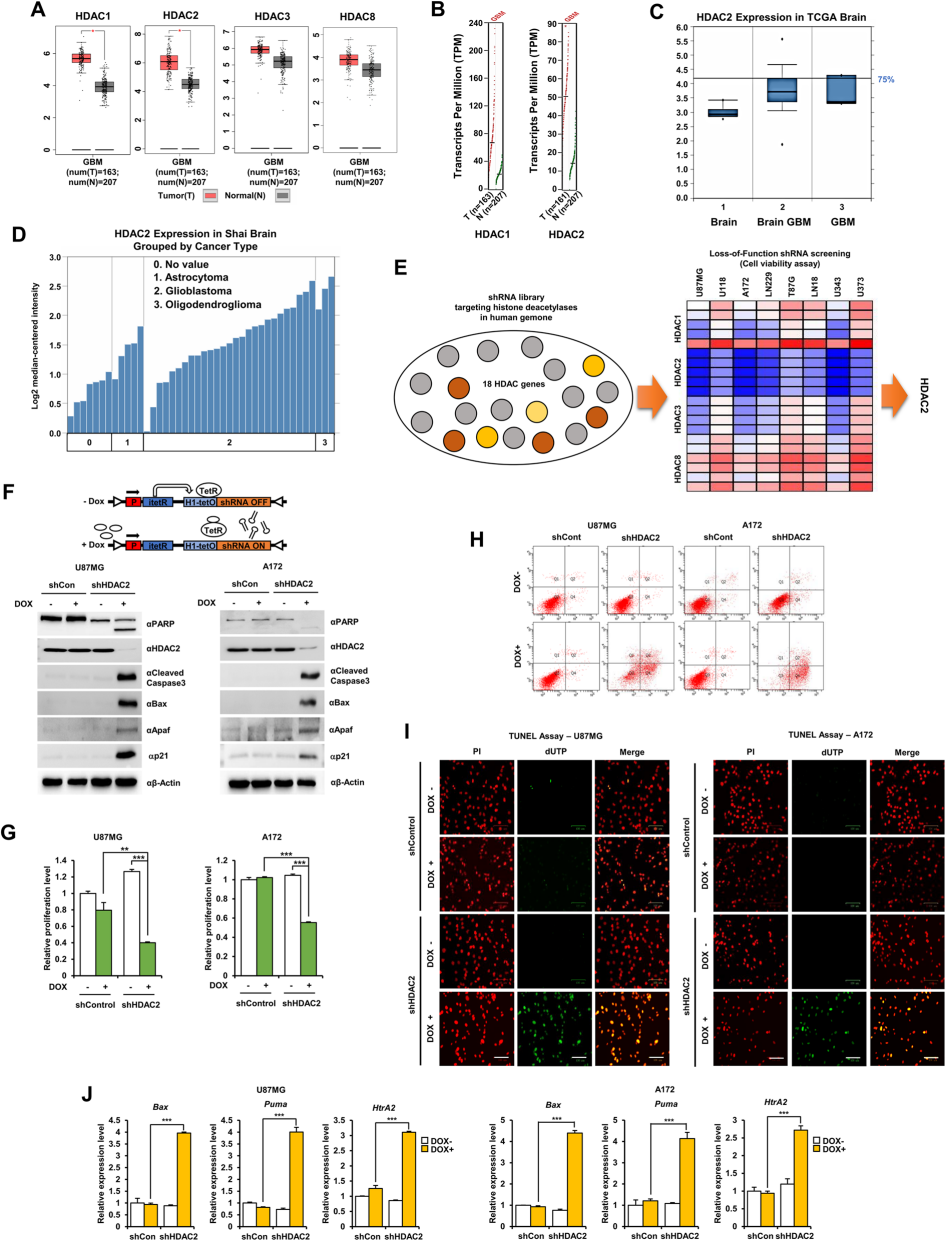
HDAC2 is highly expressed in GBM. **A** Expression of HDAC1, 2, 3 and 8 proteins. 163 GBM tissues (T) and 207 normal tissues (N) were analyzed from the GEPIA Database. *p < 0.05. **B** HDAC1 and HDAC2 expression in GBM tissues from the GEPIA Database. Axis units are Log^2^(TPM + 1) (TPM: Transcripts Per Million). **C** Box plots derived from gene expression data from the Oncomine database comparing expression of HDAC2. **D** HDAC2 expression levels from the Oncomine database. **E** Screening of a shRNA library systematically targeting HDAC genes in GBM cells. Cell viability was measured by using the MTT assay. HDAC knockdown was performed using the lentiviral system of shRNA library (five unique shRNA of HDACs) targeting 18 types of HDACs in GBM cells (U87MG, U118, A172, LN229, T98G, LN18, U343, and U373). Red: High-cell viability, Blue: Low-cell viability. **F** Model of lentiviral expression of DOX-inducible shRNA targeting human *HDAC2*. **G** Cell proliferation in DOX-inducible shHDAC2 GBM cells. **H** FACS analysis in DOX-inducible shHDAC2 GBM cells. **I** TUNEL assay in DOX-inducible shHDAC2 GBM cells. TUNEL-positive cells were stained (green), and nuclei were counterstained with PI (red). **J** mRNA expression of apoptotic cell death markers (*Bax, Puma* and *HtrA2*) using qPCR in DOX-inducible shHDAC2 GBM cells with doxycycline. Scale bar: 100 μm. All data are expressed as the mean ± SD from three independent experiments, each performed in triplicate. *p < 0.05, **p < 0.01, ***p < 0.001

We first investigated the expression of HDAC2 in human glioma patients via analysis of the GEPIA and TCGA public databases. HDAC1 and HDAC2 were significantly increased in GBM compared to normal tissues (Fig. 1A, B). Especially, HDAC2 was highly increased in GBM as indicated by TCGA Brain statistics (Fig. 1C). In the Shai Brain from TCGA database, HDAC2 was highly expressed in all brain tumors, including astrocytomas, oligodendrogliomas, and glioblastomas (Fig. 1D). Furthermore, we confirmed protein levels of class I HDACs in 8 GBM cells and normal brain cells, and HDAC2 proteins were highly expressed in GBM cells (Additional file 1: Fig. S1A and B). We also analyzed cell viability by using shRNA library (five unique shRNA) targeting 18 types of HDACs in various GBM cells, identifying HDAC2 knockdown was the most effective in promoting GBM cell death (Fig. 1E), and cell viability was also reduced to less than 50% over 6 days (Additional file 1: Fig. S1C). The knockdown of HDACs by lentiviral shRNA was measured and confirmed cell viability (Additional file 1: Fig. S1D and E). As expected, HDAC2 knockdown GBM cells underwent apoptosis more frequently than control cells. Collectively, HDAC2 is might be an essential factor in GBM tumorigenesis and could target a novel epigenetic therapeutic strategy in GBM patients.

To consider HDAC2 is necessary for GBM cell survival, we made DOX-inducible shHDAC2 GBM stable cells that expressed shRNA targeting HDAC2 upon doxycycline treatment (Additional file 1: Fig. S1F). We determined whether HDAC2 knockdown induces GBM cell death using western blot. As a result, the expression of cleaved-PARP, cleaved caspase-3, Bax, Apaf-1, and p21 increased (Fig. 1F), and cell proliferation decreased in DOX-inducible shHDAC2 GBM cells upon doxycycline treatment (Fig. 1G). We also confirmed that HDAC2 is expressed in the nucleus of GBM cells by immunofluorescence (IF) analysis (Additional file 1: Fig. S1G). GBM cell death significantly increased in DOX-inducible shHDAC2 GBM cells upon doxycycline by FACS analysis (Fig. 1H) and TUNEL assay (Fig. 1I), indicating HDAC2 knockdown increased GBM cell death.

Next, we confirmed whether HDAC2 knockdown enhanced apoptotic genes’ transcriptional activity to induce GBM cell death using qPCR. *Bax*, *Puma*, and *HtrA2* mRNA significantly increased in DOX-inducible shHDAC2 GBM cells upon doxycycline treatment (Fig. 1J). We also validated the transcriptional activity of Puma to determine the functional significance of HDAC2 using luciferase activity assay. The luciferase activity of Puma increased in HDAC2 knockdown GBM cells compared with control GBM cells or Dox-untreated shHDAC2 GBM cells (Additional file 1: Fig. S1H). Additionally, we tested whether Romidepsin, a selective HDAC1/2 inhibitor, affected anti-tumorigenic effects in GBM cells. Interestingly, the cell viability was remarkably decreased in HDAC2 knockdown GBM cells by Romidepsin treatment (Additional file 1: Fig. S1I). Taken together, HDAC2 knockdown regulates apoptosis and anti-proliferation in GBM cells, suggesting that HDAC2 plays an important role in the development and progression of GBM.

### HDAC2 knockdown GBM induces cell death by controlling the expression of miR-3189 and GLUT3

**Fig. 2:**
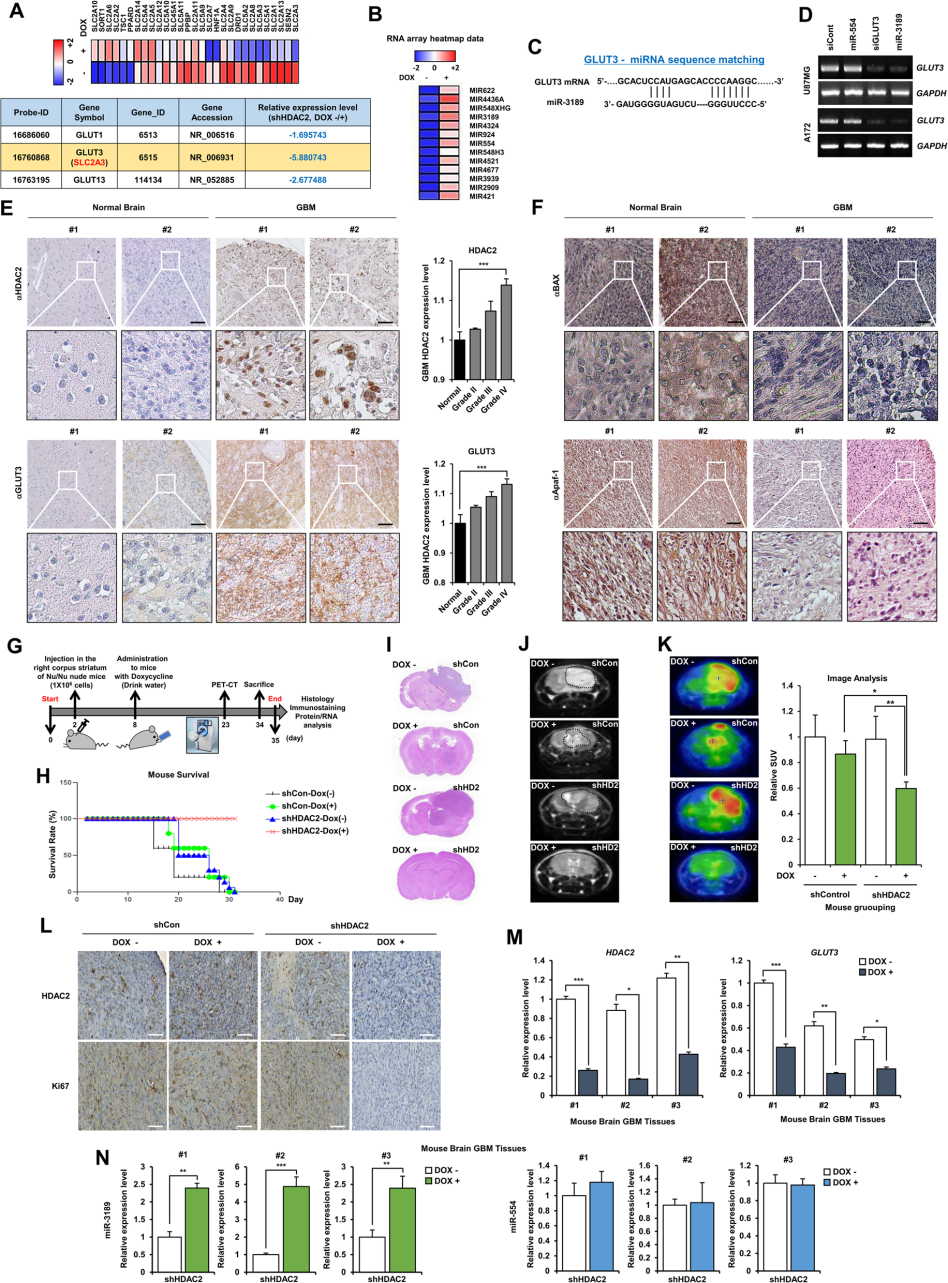
HDAC2 knockdown inhibited tumorigenesis in GBM mouse model by inhibiting miR-3189-mediated GLUT3 expression. **A** Heatmap of RNA sequence analysis in DOX-inducible shHDAC2 GBM cells w/wo doxycycline (*n* = 3). **B** Heatmap of miRNA expression in DOX-inducible shHDAC2 GBM cells w/wo doxycycline (n = 3). **C** miR-3189 binding site of GLUT3 sequences. **D** Measurement of *GLUT3* mRNA expression by qPCR. **E** IHC of human GBM tissue samples (TMA) for HDAC2 and GLUT3. Quantification of protein levels of HDAC2 and GLUT3. GBM TMA including human brain normal, grade 1, 2, 3, and 4 were classified using IHC. Scale bar: 50 μm. **F** IHC of human GBM tissue samples (TMA) for Bax and Apaf-1. Scale bar: 50 μm. **G** Schematic outline of in vivo experimental procedure. **H** Survival rate of orthotopic GBM mouse models by DOX-inducible shHDAC2 U87MG cells w/wo doxycycline. Tumorigenesis in orthotopic GBM mouse models. Brain sections of mice were stained with H&E. **J** CT imaging in orthotopic GBM mouse models. **K** PET imaging in orthotopic GBM mouse models. Graphic representation of SUV image analysis (mean area) rate of each group. **L** IHC staining of HDAC2 and Ki67 in mouse GBM tissues. Scale bar: 50 μm. **M** mRNA expression of *HDAC2* and *GLUT3* in orthotopic GBM mouse tissues by DOX-inducible shHDAC2 U87MG cells w/wo doxycycline using qPCR. **N** Expression of miR-544 and miR-3189. Representative images of human GBM and mice GBM tissue. Scale bars: 50 μm. All data are expressed as the mean ± SD from three independent experiments, each performed in triplicate. *p < 0.05, **p < 0.01, ***p < 0.001

Recently, HDAC2 silencing was reported to suppress proliferation and tumorigenesis of GBM [20], but the precise molecular mechanism is unknown. Therefore, to further investigate the physiological relevance of HDAC2 in GBM, we verified the correlation between HDAC2 knockdown and GBM cell death in HDAC2 knockdown A172 cells by RNA-sequencing analysis (Fig. 2A). HDAC2 knockdown efficiently decreased the subset of genes encoding glucose transporter proteins required for glucose metabolism, and GLUT3 was significantly downregulated. Thus, we examined whether HDAC2 knockdown inhibits GLUT3 expression by Immunofluorescence analysis, and GLUT3 expression significantly suppressed in HDAC2 knockdown GBM cells (Additional file 1: Fig. S2A). To confirm the clinical relevance, we generated Kaplan-Meier curves from “Freije”, “Vital” and “Gravendee” datasets. GLUT3 expression poorly affects the survival rate for all datasets (Additional file 1: Fig. S2B). To understand how HDAC2 knockdown suppressed GLUT3 expression and induced GBM cell death, we investigated GLUT3-targeting transcriptional regulators associated with HDAC2 expression from the RNA-sequencing dataset and analyzed miRNA gene expression profiles that regulated GLUT3 expression (Fig. 2B). We found that miR-3189 contained a complementary sequence to the GLUT3–3’UTR, which might inhibit GLUT3 expression (Fig. 2C). To verify this binding potential of miR-3189, we performed miRNA target prediction analysis using the MiRanda, and TargetScan databases. Interestingly, miR-3189-mediated GLUT3 expression has not been reported in GBM and other tumors. We also validated whether *GLUT3* mRNA expression could be regulated in miR-3189-mimics transfected GBM cells (Fig. 2D). miR-3189 strongly repressed GLUT3 transcription and siHDAC2 also showed the same results (Additional file 1: Fig. S2C). Thus, HDAC2 knockdown induced GBM cell death via miR-3189-mediated GLUT3 repression. We investigated whether the expression of HDAC2 and GLUT3 was upregulated in GBM tissues (human GBM TMA: US Biomax, Derwood, USA) using IHC. As expected, HDAC2 increased in the nucleus and GLUT3 increased in the cytoplasm in human GBM patient tissues compared to normal brain tissues (Fig. 2E). Bax and Apaf-1 decreased in human GBM patient tissues (Fig. 2F), suggesting that both HDAC2 and GLUT3 positively contribute to GBM progression.

Next, to determine whether HDAC2 knockdown meaningfully inhibit GBM progression in an in vivo preclinical mouse models as in vitro, we orthotopically xenografted DOX-inducible control and DOX-inducible shHDAC2 U87MG cells into immune-deficient BALB/C^nu/nu^ mice and administrated the drinking water containing doxycycline after 8 days from the experiment starts (Fig. 2G). We observed that while the bodyweight (Additional file 1: Fig. S2D) and survival rate (Fig. 2H) remained steady in DOX-inducible shHDAC2 U87MG-injected mice by doxycycline treatment, and rapidly decreased in DOX-untreated mice and control mice from the fourth week. Also, the tumor growth of Dox-treated shHDAC2 GBM mice was inhibited than Dox-untreated shHDAC2 GBM mice and control mice using H&E histological analysis (Fig. 2I). In addition, MRI images (Fig. 2J) and PET images (Fig. 2K) (SUV: Standardized Uptake Value; mice red image ratio) were compared by GBM tumor scans. Ki67 decreased in HDAC2 knockdown mice GBM brain upon doxycycline treatment compared to control mice brain or DOX-untreated shHDAC2 mice GBM brain using IHC analysis, indicating that HDAC2 knockdown significantly inhibited GBM tumorigenesis (Fig. 2L). Whereas Apaf-1 and Bax increased in mice GBM tissues (Additional file 1: Fig. S2E). As expected, *GLUT*3 mRNA expression decreased in HDAC2 knockdown mice GBM tissues (Fig. 2M), and HDAC2 expression inhibited in Dox-treated shHDAC2 GBM tissues (Additional file 1: Fig. S2F). Furthermore, we investigated whether miR-3189 expression was increased in DOX-treated HDAC2 knockdown mice GBM tissues upon doxycycline treatment (Fig. 2N). miR-3189 expression was significantly increased by HDAC2 knockdown, suggesting an inverse correlation between miR-3189 and GLUT3 expression in GBM. Taken together, HDAC2 regulates GBM tumorigenesis by controlling miR-3189 and GLUT3 expression.

### miR-3189 inhibits tumor growth in orthotopic mouse GBM model

**Fig. 3:**
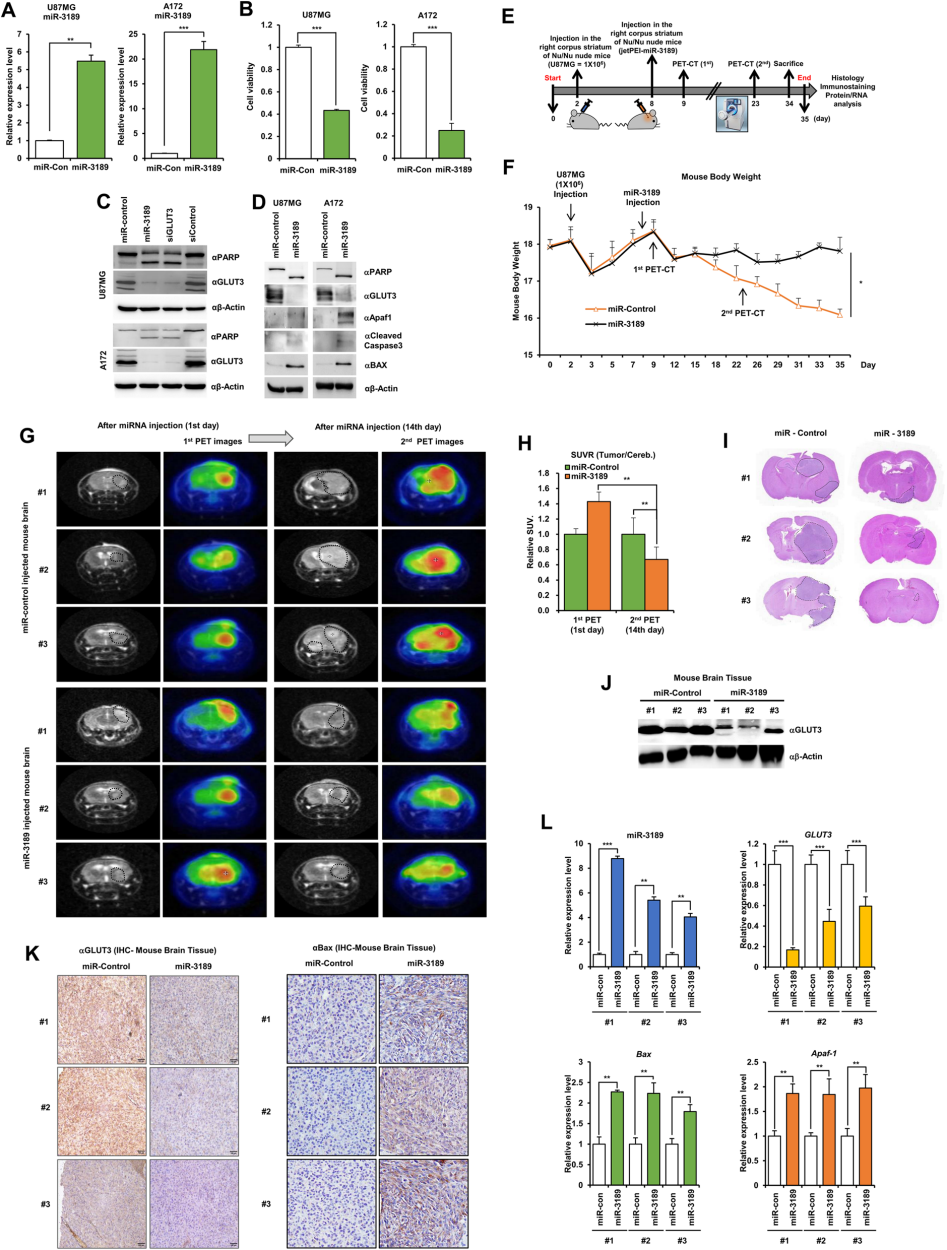
miR-3189 suppresses tumorigenesis of in vivo mouse GBM. **A** qPCR analysis of miR-3189 expression in GBM cells transfected with miR-3189 mimics. **B** Cell viability was measured in miR-3189-transfected GBM cells by MTT assay. **C** PARP and GLUT3 expression were measured in *GLUT3* siRNA- or miR-3189-transfected GBM cells by western blot. **D** Cell death markers were analyzed by western blot with indicated antibodies in miR-3189 mimic-transfected GBM cells. **E** Schematic outline of in vivo experimental procedure. **F** Mouse bodyweight of in vivo mouse GBM models. **G** PET-CT imaging in orthotopic mouse GBM models by miR-3189 transfection. **H** SUV image analysis (mean area) in miR-3189-expressing mouse GBM models. Graphic representation of SUV image analysis (mean area) rate of each group. **I** Tumorigenesis in orthotopic mouse GBM models by miR-3189 transfection. H&E staining of brain sections of mice. **J** GLUT3 expression in mouse GBM tissues. **K** IHC analysis of GLUT3 and Bax in mouse GBM tissues transfected miR-3189. Representative images of mouse GBM tissue. Scale bar: 100 μm. **L** mRNA expression of miR-3189, *GLUT3*, *Bax*, and *Apaf-1* in mouse GBM tissues transfected miR-3189. All data are expressed as the mean ± SD from three independent experiments, each performed in triplicate. *p < 0.05, **p < 0.01, ***p < 0.001

We also investigated whether miR-3189 expression decides the cell fate in GBM cells through analysis of cell viability in miR-3189-overexpressing GBM cells. miR-3189 overexpression inhibited GBM cell growth and activated cell death processes (Fig. 3A and B). Meanwhile, miR-3189-expressing or GLUT3 knockdown GBM cells increased PARP cleavage (Fig. 3C). miR-3189 expectedly suppressed GLUT3 expression and strikingly promoted the expression of Apaf-1, cleaved caspase-3, and Bax (Fig. 3D). Thus, we showed that miR-3189-mediated GBM cell death is dependent on the downregulation of GLUT3 expression.

To determine whether miR-3189 expression effectively inhibits GBM progression in orthotopic mouse models, similar to HDAC2 knockdown, we injected U87MG cells into the brain of immune-deficient BALB/C^nu/nu^ mice, and after 6 days of GBM cell injection, JetPEI-miR-3189 was directly injected in tumor sites (Fig. 3E). The next day after miR-3189 injection, we scan PET-CT images on the 1st day (1st PET-CT) and 14th day (2nd PET-CT). We observed the bodyweight of U87MG-injected mice by miR-3189 treatment (Fig. 3F) and were steadily maintained by miR-3189 treatment. Whereas miR-3189 untreated mice rapidly decreased from 24 days.

To measure the in vivo efficacy of miR-3189 on tumor growth in GBM mouse models, MRI images and PET images (Fig. 3G) were compared by tumor scans at 0 days and 14 days after miRNA injection. SUV ratio of the red image in mice brain highly increased in miR-control treated mice but not in miR-3189 treated mice (Fig. 3H), indicating that miR-3189 effectively inhibited GBM tumorigenesis. Also, the tumor growth in U87MG injected mice by miR-3189 treatment was inhibited more than miR-control treatment by H&E histological analysis (Fig. 3I). GLUT3 expression was confirmed by western blot (Fig. 3J) and has significantly decreased in U87MG-injected mice brain tissues upon miR-3189 treatment compared to miR-3189 untreated mice using IHC analysis. Bax increased by miR-3189 treatment (Fig. 3K). Additionally, we validated mRNA expression of miR-3189, *GLUT3*, *Bax*, and *Apaf-1* upon miR-3189 treatment using qPCR. As previous results, GLUT3 mRNA expression decreased in miR-3189-treated U87MG-injected mice brain, whereas the mRNA expression of *Bax* and *Apaf-1* increased (Fig. 3L). Therefore, miR-3189 significantly decreased GBM tumorigenesis by targeting GLUT3 expression in GBM mouse models.

### miR-3189 induced GBM cell death via the transcriptional repression of GLUT3

**Fig. 4:**
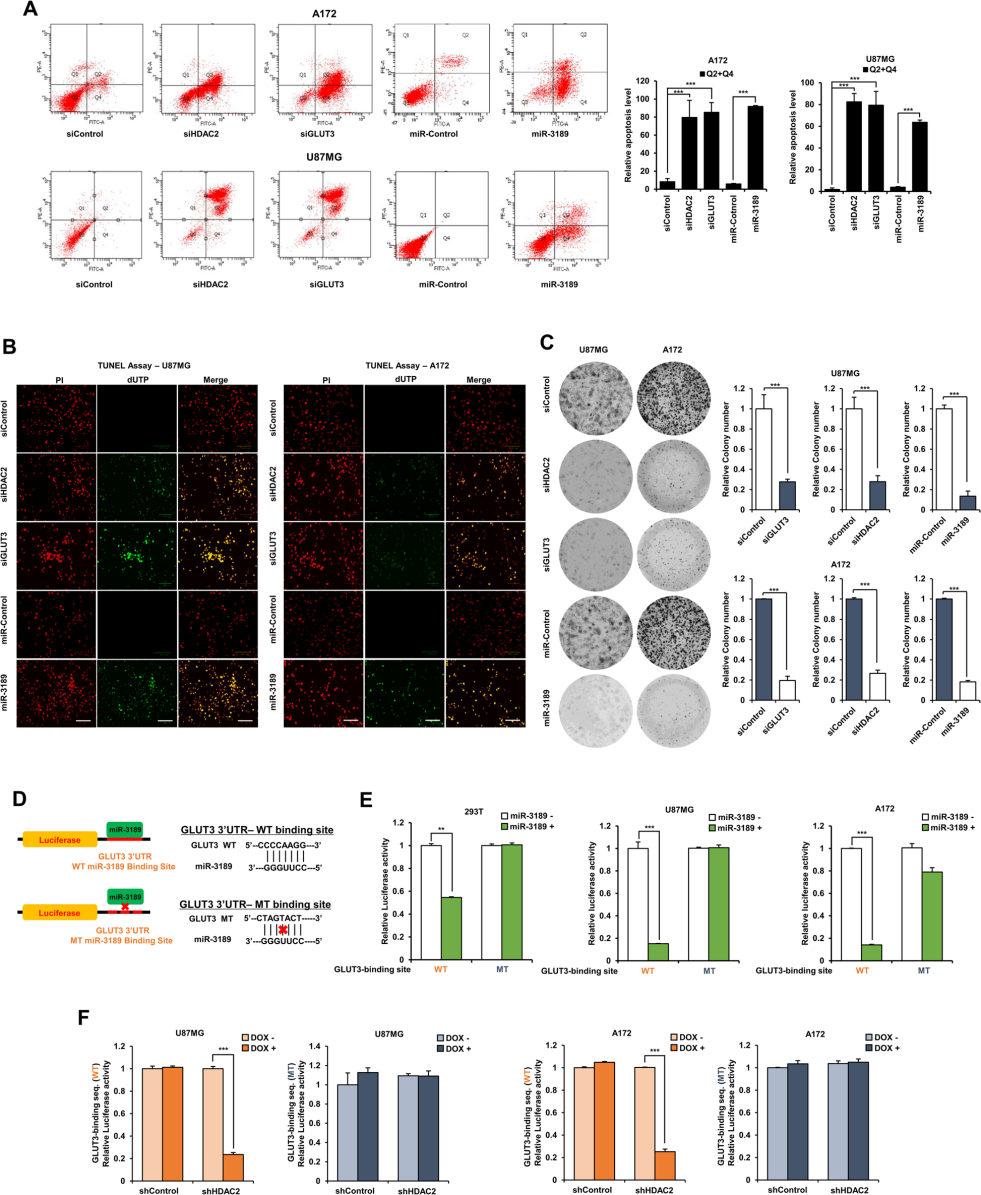
GLUT3 knockdown by miR-3189 increased cell death of GBM cells. **A** FACS analysis of GBM cells expressing control siRNA, *HDAC2* siRNA, *GLUT3* siRNA, control miRNA, and miR-3189 mimics. **B** TUNEL assay in *HDAC2* siRNA, *GLUT3* siRNA, control miRNA, and miR-3189 mimics-transfected GBM cells. TUNEL-positive cells: green, Nuclei with PI: red, Merged cells: yellow. Scale bar: 100 μm. **C** Colony formation assay of miR-3189 mimic-transfected GBM cells. Quantification of colony formation. **D** Schematic of miR-3189 and *GLUT3* 3′-UTR binding site interactions. **E** Measurement of luciferase reporter activity of pmirGLO-GLUT3-Luc for binding of miR-3189. pmirGLO-GLUT3MT-Luc plasmid were transiently transfected w/wo miR-3189 mimics into 293 T and GBM cells. Reporter activities were normalized relative to Renilla luciferase activities. **F** Luciferase reporter assays using pmirGLO-GLUT3MT-Luc plasmid transiently transfected into DOX-inducible shHDAC2 GBM cells. Reporter activities were normalized relative to Renilla luciferase activities. All data are expressed as the mean ± SD from three independent experiments, each performed in triplicate. **p < 0.01, ***p < 0.001

GLUT3 is the essential glucose transporter involved in brain glucose uptake, and its role is well-documented in GBM metabolism. First, we observed GBM survival in GLUT3 knockdown GBM cells by FACS analysis. GLUT3 knockdown induced GBM cell death similar to HDAC2 knockdown or miR-3189 overexpression, and the frequency of apoptosis by FACS analysis was increased in all early (Q2) and late (Q4) stages (Fig. 4A). In addition, we confirmed that the apoptotic cells increased upon GLUT3 knockdown using TUNEL assay (Fig. 4B), and GLUT3 knockdown significantly decreased the colony formation of GBM cells compared to control GBM cells, and miR-3189 also showed the same results (Fig. 4C).

We analyzed whether GLUT3 knockdown or miR-3189 overexpression increased cell death markers’ expression using qPCR. As expected, Pro-apoptosis genes increased in GLUT3 knockdown GBM cells and miR-3189-expressing GBM cells (Additional file 1: Fig. S3A and B). Besides, to validate that miR-3189 directly regulated GLUT3, we performed luciferase reporter assays with miR-3189 mimics and pmirGLO plasmids bearing wild-type or mutant GLUT3 3′-UTR sequences of putative miR-3189 binding sites (Fig. 4D). These results showed that miR-3189 dramatically repressed luciferase activity of pmirGLO-GLUT3wt containing the miR-3189 binding site from the wild type GLUT3 3′-UTR; however, luciferase activity upon pmirGLO-GLUT3mt did not repress by miR-3189 in 293 T and GBM cells (Fig. 4E), indicating that miR-3189 can significantly inhibit GLUT3 expression via binding to GLUT3 3′-UTR. Because HDAC2 knockdown was highly influential in inducing GBM cell death to provide the direct evidence that HDAC2 knockdown induced GLUT3-mediated cell death via miR-3189 upregulation, we investigated the luciferase activity of pmirGLO-GLUT3wt in HDAC2 knockdown GBM cells by doxycycline treatment (Fig. 4F). HDAC2 knockdown effectively decreased the luciferase activity of pmirGLO-GLUT3wt to induce GBM cell death and remained ineffective in repressing luciferase activity in pmirGLO-GLUT3mt-expressing GBM cells. These results suggest that HDAC2 knockdown increased GBM cell death via inhibition of miR-3189-mediated GLUT3 expression.

### HDAC2 repression controls the metabolism and proliferation of GBM

**Fig. 5:**
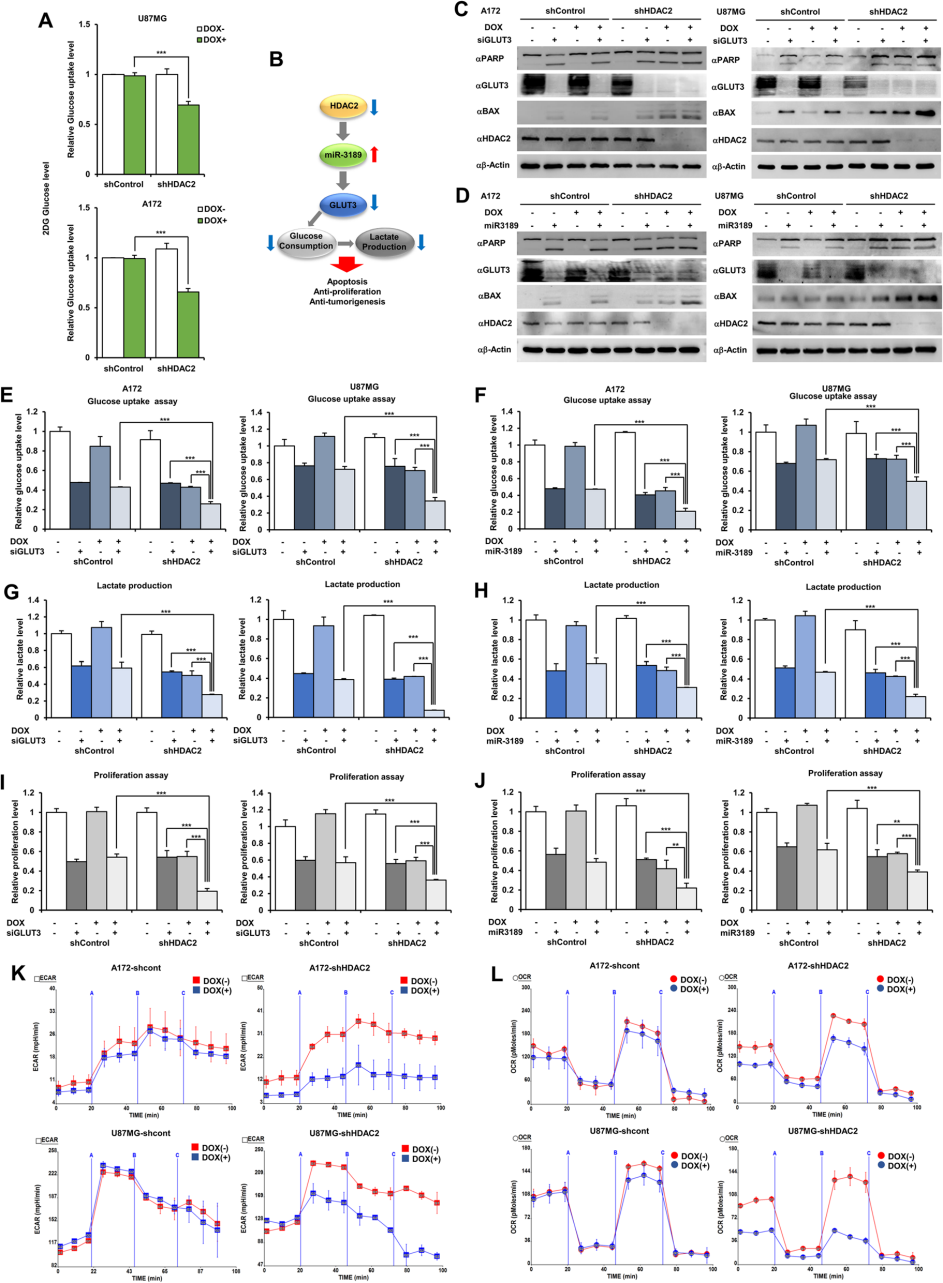
miR-3189 regulates glucose metabolism through GLUT3 inhibition in HDAC2 knockdown GBM cells. **A** Glucose uptake in DOX-inducible shHDAC2 GBM cells. **B** Metabolism schematic diagram of HDAC2 knockdown GBM cells. **C** Cell death analysis in *GLUT3* siRNA-transfected DOX-inducible shHDAC2 GBM cells w/wo doxycycline by using western blot with indicated antibodies. **D** Cell death analysis in miR-3189 mimic-transfected DOX-inducible shHDAC2 GBM cells w/wo doxycycline by using western blot with indicated antibodies. **E-F** Glucose uptake in DOX-inducible shHDAC2 GBM cells using *GLUT3* siRNA (E) or miR-3189 mimics (F). **G-H** Lactate production in DOX-inducible shHDAC2 GBM cells using transfected with either *GLUT3* siRNA (G) or miR-3189 mimics (H). **I-J** Cell proliferation of DOX-inducible shHDAC2 GBM cells using *GLUT3* siRNA (I) or miR-3189 mimics (J). **K-L** Mitochondrial versus non-mitochondrial metabolism in DOX-inducible shHDAC2 GBM cells w/wo doxycycline. Time-dependent Oxygen Consumption Rate (OCR, K) measurements and Extracellular Acidification Rate (ECAR, L) measurements were traced with a Seahorse Bioscience XF96 analyzer. Data are expressed as the mean ± SD for triplicates. One-way ANOVA with multiple comparisons correction (*p* < 0.001). All data are expressed as the mean ± SD from three independent experiments, each performed in triplicate. **p < 0.01, ***p < 0.001

In many studies, the reduced glucose uptake and lactate production in glucose metabolism has been known to inhibit tumor cell viability.^15^ Therefore, to consider whether cell death and proliferation in GBM cells might correlate with glucose metabolism, we examine that the repression of GLUT3 by HDAC2 knockdown induces GBM cell death via inhibition of glucose metabolism. Glucose uptake significantly decreased in DOX-inducible shHDAC2 GBM cells with doxycycline treatment (Fig. 5A) and confirmed the same effect in the *HDAC2* siRNA treatment (Additional file 1: Fig. S4A). Indeed, HDAC2 knockdown resulted in reduced glucose uptake and lactate production in GBM cells (Additional file 1: Fig. S4B). Therefore, our results strongly propose that HDAC2 might serve as a master regulator of GBM cell death via regulation of glucose metabolism by miR-3189-mediated GLUT3 expression (Fig. 5B).

We next sought to confirm the functional relevance of reduced glucose metabolism and GBM cell death. Many studies reported that GLUT3 is highly expressed in GBM and contributes to the growth of brain tumors [17]. Because miR-3189 has not been investigated in all cancer-contained brain tumors, to assess the importance of the apoptotic effect of GLUT3 knockdown and miR-3189 expression, we transfected *GLUT3* siRNA or miR-3189 mimics into DOX-inducible shHDAC2 GBM cells. Our results definitively show that *GLUT3* siRNA-transfected shHDAC2-expressing GBM cells significantly increased PARP cleavage and Bax compared with DOX-inducible control GBM cells or DOX-untreated shHDAC2 GBM cells. Equally important, miR-3189-transfected shHDAC2-expressing GBM cells increased GBM cell death via downregulation of GLUT3 (Fig. 5C and D), suggesting that HDAC2 expression-dependent cell death might occur by miR-3189-mediated GLUT3 inhibition. We also investigated cellular proliferation by expressing *GLUT3* siRNA, miR-3189 mimics, and *HDAC2* siRNA in GBM cells, confirming that both GLUT3 knockdown and miR-3189 overexpression decreased GBM proliferation, similar to HDAC2 knockdown (Additional file 1: Fig. S4C).

To demonstrate whether GLUT3 downregulation reduced glucose uptake and lactate production to cause GBM cell death, we measured glucose uptake, lactate production, and cell proliferation in DOX-inducible shHDAC2 GBM cells. *GLUT3* siRNA or miR-3189 mimics synergistically decreased the glucose uptake (Fig. 5E and F) and lactate production (Fig. 5G and H) in shHDAC2-expressing GBM cells than individually transfected GBM cells, but the change of glucose uptake and lactate production did not observe in GLUT1 or GLUT2 knockdown GBM cells (Additional file 1: Fig. S4D and E). Similarly, the cellular proliferation was decreased in the same condition (Fig. 5I and J), suggesting that the combined treatment of *GLUT3* siRNA or miR-3189 remarkably reduced these metabolic changes and cellular proliferation in HDAC2 knockdown GBM cells. We also investigated the Extracellular Acidification Rate (ECAR) and Oxygen Consumption Rate (OCR) in DOX-inducible shHDAC2 GBM cells with doxycycline using a Seahorse extracellular flux analyzer. Both ECAR and OCR significantly decreased in HDAC2 knockdown GBM cells (Fig. 5K) but not in control GBM cells (Fig. 5L). These results strongly support that HDAC2 knockdown was directly associated with metabolite regulation in mitochondrial respiration and glycolysis via GLUT3 inhibition and ultimately induced GBM cell death.

### HDAC2 knockdown increases cell death and decreases tumor-sphere formation in GSCs

Most GBM consists of mixed glioma cells and glioma stem cells (GSCs) associated with tumorigenesis and resistance to common therapies in GBM [6]. GSCs also have tumor-initiating, self-renewing properties and the unique ability to grow in microenvironments with limited nutrients [17]. GSCs can promote cancer recurrence and drug resistance by evading cell death [7, 12]. Thus, the discovery of target genes and metabolites characteristic of GSCs is an essential step to enhance apoptosis in designing therapeutic strategies to treat GBM.

**Fig. 6:**
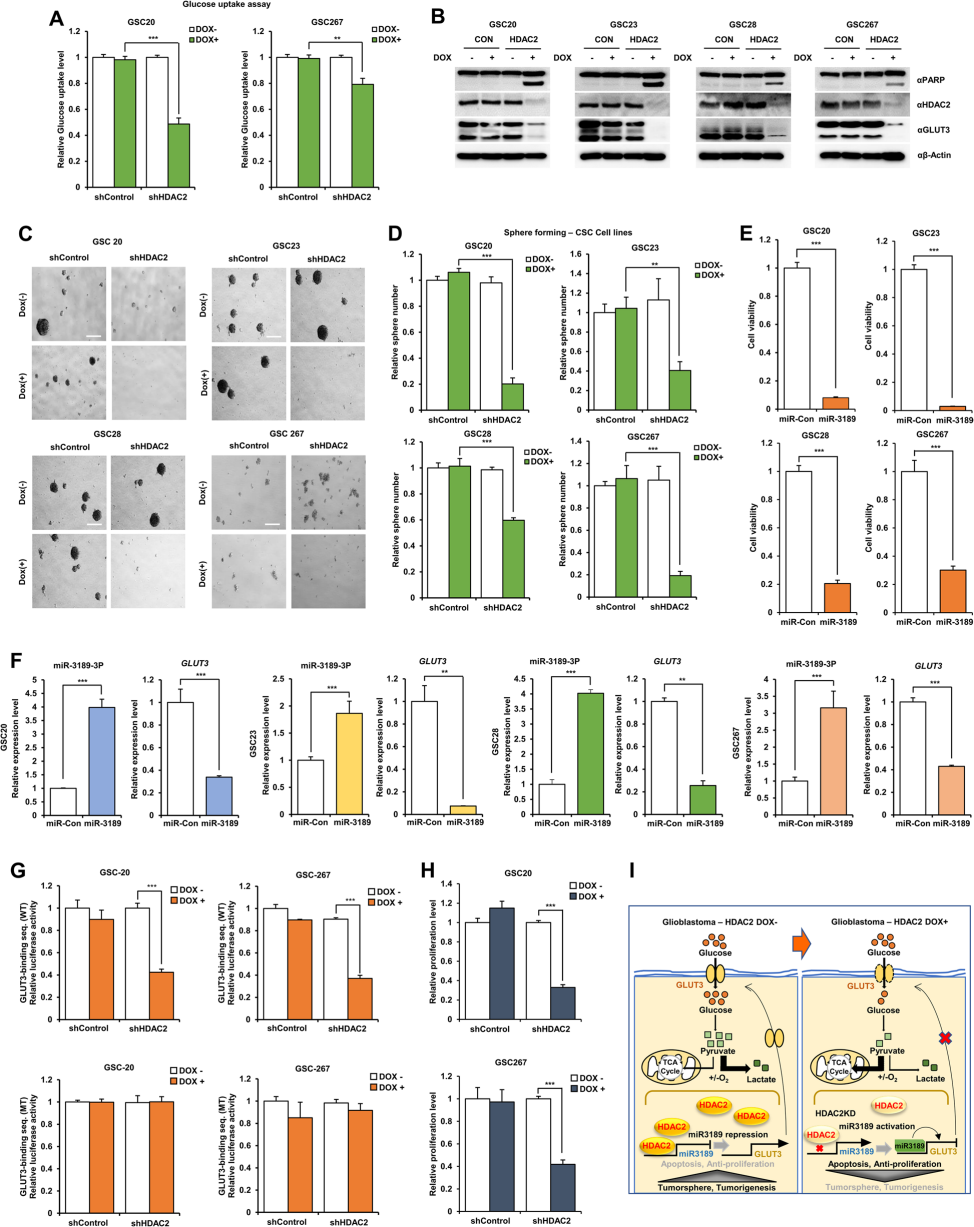
HDAC2 knockdown inhibits tumor-sphere formation and proliferation via increasing miR-3189-mediate GLUT3 in GSCs. **A** Glucose uptake in DOX-inducible shHDAC2 GSCs. **B** PARP cleavage in DOX-inducible shHDAC2 GSCs w/wo doxycycline was analyzed using western blot with indicated antibodies. **C** Tumor-sphere formation assay in GSCs. Cell picture images were taken at 40X. Scale bar: 400 μm. **D** Quantification of tumor-sphere number. **E** Cell viability in miR-3189-transfected GSCs by WST-8 assays. **F** mRNA expression of *GLUT3* in miR-3189-expressing GSCs using qPCR. **G** Luciferase reporter activity in DOX-inducible shHDAC2 GSCs using dual-luciferase assay. GSCs were transiently transfected with pmirGLO-GLUT3MT-Luc. Reporter activities were normalized relative to *Renilla* luciferase activities. Reporter activities were expressed as the mean ± SD for triplicates. **H** Cell proliferation of shHDAC2 expressing GSCs. **I** Graphical conclusion of the mechanism of cell death in HDAC2 knockdown GBM/GSCs. All data are expressed as the mean ± SD from three independent experiments, each performed in triplicate. *p < 0.05. **p < 0.01, ***p < 0.001

To understand these most aggressive and therapeutic-resistant GBM cells, we analyzed the role of HDAC2 in regulating cell death by inhibiting miR-3189-mediated GLUT3 in DOX-inducible shHDAC2 GSCs. HDAC2 knockdown GSCs (GSC20, GSC23, GSC28, and GSC267) significantly decreased glucose uptake levels (Fig. 6A) and increased cleaved PARP (Fig. 6B) upon doxycycline treatment, these results displayed the same results in GBM cells. Importantly, HDAC2 knockdown also inhibited the tumor-sphere formation of GSCs (Fig. 6C and D), indicating that the survival of GSCs was directly regulated by HDAC2 expression level. Thus, we expected that GSCs would be highly sensitive to miR-3189 expression that inhibits GLUT3 expression. We also assessed the cell death effect in miR-3189-expressing GSCs. miR-3189 overexpression dramatically decreased the cell viability of GSCs (Fig. 6E). Indeed in these results, overexpressing miR-3189 repressed GLUT3 transcription in GSCs (Fig. 6F), suggesting that inhibition of miR-3189-mediated GLUT3 reduced tumor growth and cell viability not only in GBM cells but in GSCs.

To further determine whether the direct binding of miR-3189 to the GLUT3 3′-UTR region in GSCs, we observed the transcriptional activity of GLUT3 in pmirGLO-GLUT3wt or pmirGLO-GLUT3mt transfected DOX-inducible shHDAC2 GSCs by using luciferase assay. HDAC2 knockdown GSCs upon doxycycline treatment significantly decreased luciferase activity of GLUT3wt 3′-UTR, but not GLUT3mt 3′-UTR (Fig. 6G), and decreased GSCs proliferation (Fig. 6H). Additionally, we confirmed whether miR-3189 affects the gene expression of HDAC2 or other miRNAs (Additional file 1: Fig. S5A and B). Both expressions of HDAC2 and miRNAs did not change by miR-3189, expecting miR-3189 is involved in the downstream regulatory pathway of HDAC2. These results show that HDAC2 knockdown increased miR-3189 expression which was also recruited to the GLUT3 3′-UTR to inhibit GLUT3 expression, suggesting that HDAC2 knockdown inhibits tumorigenesis and GSC-sphere formation by inducing GSC cell death via miR-3189-mediated GLUT3 downregulation (Fig. 6I). Collectively, HDAC2 is a critical GBM/GSC progression marker and an ideal candidate for targeted therapy.

## Discussion

Several previous studies have shown that histone acetylation and deacetylation regulates gene transcription rates in the central nervous system via epigenetic modification, which can affect brain development and memory, and abnormally controls in Alzheimer’s disease, Huntington’s disease, and Parkinson’s disease, as well as other brain diseases [21]. HDAC2 has been reported to be highly expressed in AD mouse models, which are also known to have abnormal histone acetylation and reduced transcription [22, 23]. In the brain, HDAC2 is known to play a significant role in the developmental stage and neuronal differentiation [24, 25].

In many cancers, including GBM, HDACs are essential for regulating cancer progression [26, 27]. The current study provides insight into the mechanism by which inhibition of HDACs enhances apoptosis, similar to anticancer agents in cancer cells [28]. Treatment of GBM cells with HDAC inhibitors can increase the sensitivity of cancer cells to chemotherapy drugs and play a significant role in inhibiting cancer growth [29]. However, it is not yet clear how the HDAC1/2 functions to suppress cell proliferation in GBM. Thus, we showed that HDAC2 is highly expressed in GBM cells, and HDAC2 knockdown in GBM cells causes metabolic dysfunction. In previous studies, we confirmed that HDAC2 inhibition attenuated the growth of GBM cells. To further clarify our findings, we demonstrated that HDAC2 knockdown could suppress GBM tumors in mice orthotopic xenografts that had been injected with DOX-inducible shHDAC2 U87MG after doxycycline administration. Therefore, HDAC2 knockdown GBM similarly induced cell death not only in vitro but also in vivo.

The GLUT protein family plays a vital role in metabolite uptake as intracellular glucose transporters. The GLUT3 are generally known to be highly expressed within GBM. This highly expressed GLUT3 is expected to accelerate GBM growth by supplying glucose to various GBM cells. We observed that downregulation of GLUT3 leads to abnormal metabolism, inhibited cell growth, and increased cell death in GBM. miRNAs are known to carry out tumor-suppressor functions, leading to apoptosis and reducing cell growth and survival. Most miRNAs bind to the 3′-UTR of target genes, which is untranslated, and some miRNAs sufficiently affects cell death in GBM [12, 20]. In this study, we conducted the correlation of miR-3189 and GLUT3 in GBM cells. As a result of miR-3189 treatment, we confirmed that glucose metabolism could be affected by GLUT3 downregulation. The use of miR-3189 proved the effective treatment with simultaneous HDAC inhibitors, currently used as chemotherapy drugs. Also, *HDAC2* siRNA, *GLUT3* siRNA, and miRNA mimic transfection induced similar apoptosis in GBM and GSCs. Besides, HDAC2 knockdown dramatically reduced the capacity of tumor-sphere formation in HDAC2 knockdown GSCs and induced apoptosis by caspase-3, Bax, and Apaf-1. Glucose in brain cells is an essential nutrient for energy formation through the TCA cycle and glycolytic lactate production. HDAC2 knockdown significantly reduced glucose-related metabolism by OCR analysis. Glucose uptake and lactate production reached similar conclusions, too. Therefore, for effective GBM therapy, it is necessary to develop a therapeutic agent by discovering new target factors to improving the survival rate of GBM patients through the study of cancer metabolism and epigenetic regulatory mechanisms.

## Conclusions

In summary, our findings demonstrated that HDAC2 knockdown induced cell death in GBM by controlling miR-3189 expression, repressing *GLUT3* mRNA transcription, and regulating glucose metabolism, suggesting that Therapeutic targeting of HDAC2 has the potential to restore drug sensitivity in GBM. Thus, treatment with selective inhibitors of HDAC2 could be effective in combining chemotherapy in inducing GBM/GSC cell death.

## Supplementary Information

**Additional file 1: Supplementary Fig. 1. A** Expression of Class 1 HDAC Genes and Proteins in GBM cells. GBM cells were extracted in lysis buffer, and cell lysates were analyzed using western blot analysis with class I HDAC antibodies (HDAC1, 2, 3 and 8). **B** Quantification of protein level of class I HDAC expression. Densitometric quantification of protein signals was quantified by ImageJ (Java 1.8.0_112, NIH, Bethesda, MD, USA), and the level of protein expression was normalized to β-actin. Data represent the means ± SD from three independent experiments. **C** Lentiviral infection of *HDAC2* shRNA in GBM cells. A172 and U87MG cells were incubated for 6 days post-infection prior to measurement of cell viability by using MTT assay. All data are expressed as the mean ± SD for triplicates. **D** Class I HDAC siRNA was transfected into GBM cells. Cell lysates were analyzed by western blot using the indicated antibodies. **E** Class I HDAC siRNA was transfected into GBM and normal brain cells. SVGp12 and GBM cells were incubated for 48 h. Cell viability was measured via MTT assay. **F** qRT-PCR analysis and Immunoblot analysis of HDAC2 and GLUT3 expression in control GBM cells and HDAC2^KD^ GBM cells upon doxycycline treatment. **G** IF in DOX-inducible shcontrol GBM cells and DOX-inducible shHDAC2 GBM cells upon doxycycline treatment (2.5 μg/ml) (DAPI: blue and FITC-HDAC2: green). Scale bar: 100 μm. **H** Luciferase reporter activities of DOX-inducible control and DOX-inducible shHDAC2 GBM cells (Upper: U87MG and Bottom: A172) with doxycycline. Cells were transiently transfected with reporter pGL3-Luc or pGL3-Puma-Luc plasmids. Dual luciferase activity was measured (420 nm) in cell lysates. Reporter activities were normalized relative to Renilla luciferase activities. **I** Cell viability of DOX-inducible shcontrol and DOX-inducible shHDAC2 GBM cells (Left: U87MG and Right: A172). GBM cells upon doxycycline treatment were measured in the presence or absence of Romidepsin by using WST-8 assay. All data are expressed as the mean ± SD from three independent experiments, each performed in triplicate. ***p < 0.001. **Supplementary Fig. 2. A** IF analysis of GLUT3 expression in DOX-induced shHDAC2 GBM cells and control GBM cells (DAPI: nuclei, Texas Red: GLUT3). Scale bar: 100 μm. **B** Kaplan-meier analysis of the Freije, Vital and Gravendeel dataset for SLC2A3 (GLUT3) expression. (*P* = 0.05). mRNA expression of **GLUT***3* and miR-3189 in control and *HDAC2* siRNA-transfected GBM cells. **C** mRNA expression of *GLUT3* and miR-3189 in control and *HDAC2* siRNA-transfected GBM cells. **D** HDAC2 expression in Orthotropic Brain Tumor Mouse Models. Body weight of orthotropic xenograft mouse brain tumor models (DOX-inducible shControl U87MG and DOX-inducible shHDAC2 U87MG) with or without doxycycline treatment. **E** IHC staining of apoptotic cell death markers (Apaf-1 and Bax) in normal tissues and GBM tissues of mouse brain. **F** HDAC2 expression was measured in DOX-inducible shControl U87MG cells and DOX-inducible shHDAC2 U87MG cells orthotopically injected into mouse brains. Mice were given doxycycline (2 μg/ml) in drinking water. **Supplementary Fig. 3.** Quantitative PCR Analysis of mRNA Expression of *GLUT3* and Cell Death Markers in Both *GLUT3* siRNA- and miR-3189-transfected GBM Cells. **A** mRNA expression of *GLUT3* by qRT-PCR in *GLUT3* siRNA and miR-3189 mimic-transfected GBM cells. **B** mRNA expression of apoptotic cell death markers by qRT-PCR in control and *GLUT3* siRNA and miR-3189 mimic-transfected GBM cells. All Data are expressed as the mean ± SD for triplicates. *p < 0.05, **p < 0.01, ***p < 0.001. **Supplementary Fig. 4.** Knockdown HDAC2 and GLUT3 Inhibits Glucose Metabolism and Cell Proliferation in GBM Cells. **A** Glucose uptake assay in GBM cells transfected with control siRNA or *HDAC2* siRNA. **B** Metabolite measurement of DOX-inducible shHDAC2 GBM cells with or without doxycycline treatment. Left: Glucose uptake assay, Right: Lactate production assay. **C** Cell proliferation in *HDAC2* siRNA-, *GLUT3* siRNA-, or miR-3189-transfected GBM cells. Left: A172 cells, Right: U87MG cells. **D** Glucose uptake assay of GBM cells that were transfected with either *GLUT1* siRNA or *GLUT2* siRNA. Left: U87MG cells, Right: A172 cells. **E** Lactate production assay of GBM cells that were transfected with either *GLUT1* siRNA or *GLUT2* siRNA. Left: U87MG cells, Right: A172 cells. All data are expressed as the mean ± SD for triplicates. **p < 0.01, ***p < 0.001. **Supplementary Fig. 5.** Quantitative PCR Analysis of Selected miRNA in miR-3189-transfected GSCs. **A** *HDAC2* mRNA expression in miR-3189-transfected GSCs (GSC20, GSC23, GSC28 and GSC267) after incubation for 48 h. Expression of *HDAC2* mRNA was analyzed using qRT-PCR. **B** Expression of selected miRNAs by microarray analysis in miR-3189-transfected GSCs after incubation for 48 h. Expression of miRNAs was analyzed using qRT-PCR. All data are expressed as the mean ± SD for triplicates.

**Additional file 2: Supplementary Table 1.** List of primer for qRT-PCR. **Supplementary Table 2.** Complete list of materials and reagents for experimental.

## Data Availability

All datasets generated in this work have been deposited to Gene Expression Omnibus (GEO) under accession number GSE158355, available at https://www.ncbi.nlm.nih.gov/geo/query/ acc.cgi?acc = GSE158355.

## Abbreviations

GBM: Glioblastoma
HDACs: Histone deacetylases
HDAC2: Histone deacetylase 2
SIRT: Sirtuin
TSA: Trichostatin A
GLUT: Glucose transporters
GLUT3: Glucose transporter 3
HDAC2 shRNA: DOX-inducible shHDAC2
pLKO/TetON shControl: DOX-inducible shcontrol
IF: Immunofluorescence
PET: Positron Emission Tomography
MRI: Magnetic Resonance Imaging
VOI: Volume of interest
SUV: Standard uptake value
ECAR: Extracellular Acidification Rate
OCR: Oxygen Consumption Rate
GSCs: Glioma stem cells
DOX: Doxycycline
IHC: Immunohistochemical staining
FACS: Fluorescence-activated cell sorting
qRT-PCR: Quantitative reverse transcription polymerase chain reaction
DMEM: Dulbecco’s Modified Eagle Medium
FBS: Fetal Bovine Serum
PBS: Phosphate-buffered saline
SDS: Sodium dodecyl sulfate
TCGA: The Cancer Genome Atlas
GEPIA: Gene Expression Profiling Interactive Analysis
TMA: Tissue microarray
ANOVA: Analysis of variance
