# Supplementary material for: miR-3189-targeted GLUT3 repression by HDAC2 knockdown inhibits glioblastoma tumorigenesis through regulating glucose metabolism and proliferation
Source: J Exp Clin Cancer Res. 2022 Mar 8;41:87. doi: 10.1186/s13046-022-02305-5 (PMC8903173; doi:10.1186/s13046-022-02305-5)
Supplement: Supplementary file 1 — Additional file 1: Supplementary Fig. 1. A Expression of Class 1 HDAC Genes and Proteins in GBM cells. GBM cells were extracted in lysis buffer, and cell lysates were analyzed using western blot analysis with class I HDAC antibodies (HDAC1, 2, 3 and 8). B Quantification of protein level of class I HDAC expression. Densitometric quantification of protein signals was quantified by ImageJ (Java 1.8.0_112, NIH, Bethesda, MD, USA), and the level of protein expression was normalized to β-actin. Data represent the means ± SD from three independent experiments. C Lentiviral infection of HDAC2 shRNA in GBM cells. A172 and U87MG cells were incubated for 6 days post-infection prior to measurement of cell viability by using MTT assay. All data are expressed as the mean ± SD for triplicates. D Class I HDAC siRNA was transfected into GBM cells. Cell lysates were analyzed by western blot using the indicated antibodies. E Class I HDAC siRNA was transfected into GBM and normal brain cells. SVGp12 and GBM cells were incubated for 48 h. Cell viability was measured via MTT assay. F qRT-PCR analysis and Immunoblot analysis of HDAC2 and GLUT3 expression in control GBM cells and HDAC2KD GBM cells upon doxycycline treatment. G IF in DOX-inducible shcontrol GBM cells and DOX-inducible shHDAC2 GBM cells upon doxycycline treatment (2.5 μg/ml) (DAPI: blue and FITC-HDAC2: green). Scale bar: 100 μm. H Luciferase reporter activities of DOX-inducible control and DOX-inducible shHDAC2 GBM cells (Upper: U87MG and Bottom: A172) with doxycycline. Cells were transiently transfected with reporter pGL3-Luc or pGL3-Puma-Luc plasmids. Dual luciferase activity was measured (420 nm) in cell lysates. Reporter activities were normalized relative to Renilla luciferase activities. I Cell viability of DOX-inducible shcontrol and DOX-inducible shHDAC2 GBM cells (Left: U87MG and Right: A172). GBM cells upon doxycycline treatment were measured in the presence or absence of Romidepsin by using WST-8 assay. All data a [file 13046_2022_2305_MOESM1_ESM.pdf]

Supplementary Figures

miR-3189-targeted GLUT3 repression by HDAC2 knockdown inhibits glioblastoma tumorigenesis through regulating glucose metabolism and proliferation

Kwak et al.

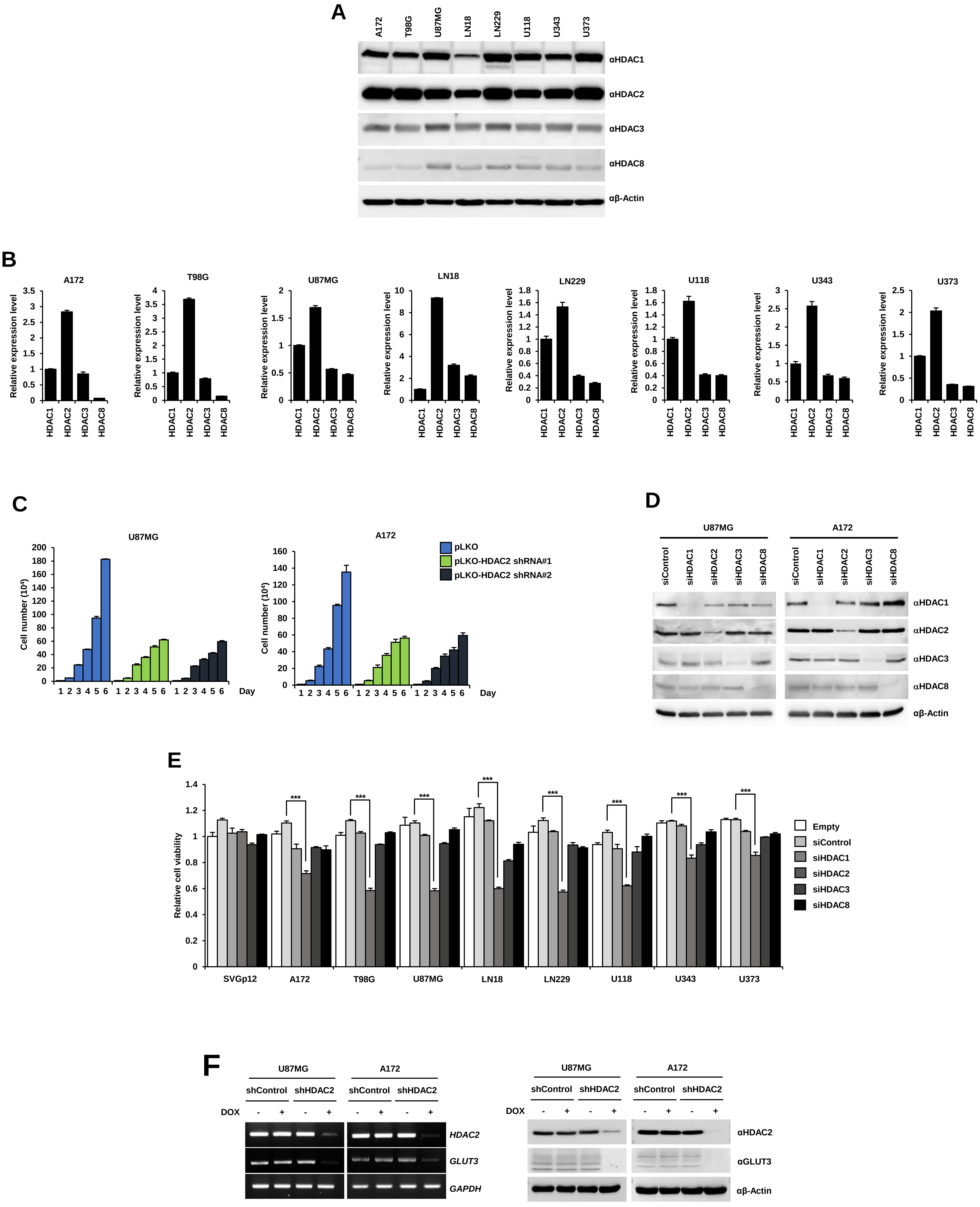

Supplementary Figure 1.

G

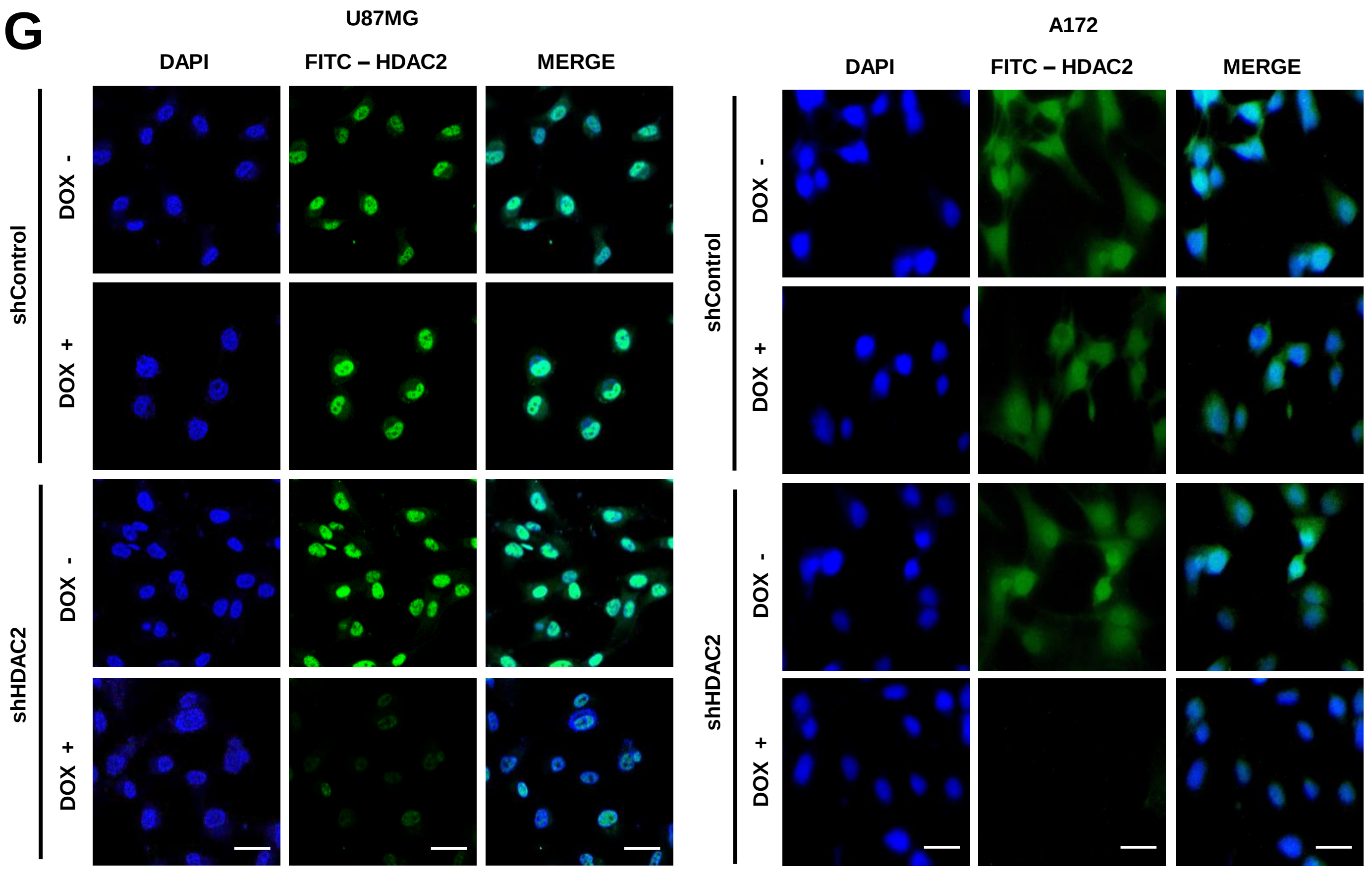

H

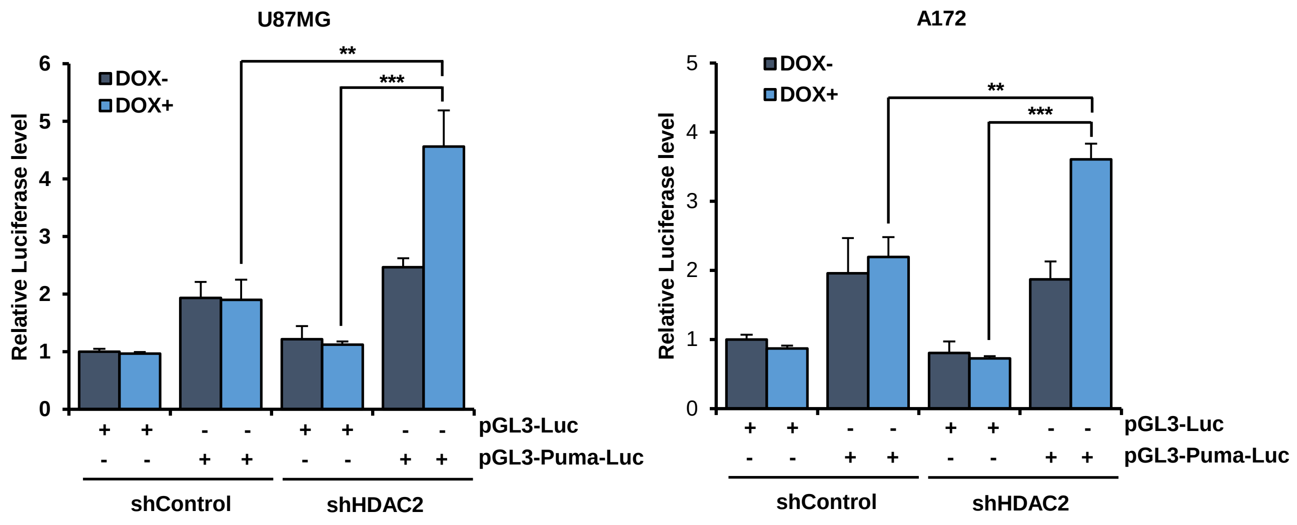

I

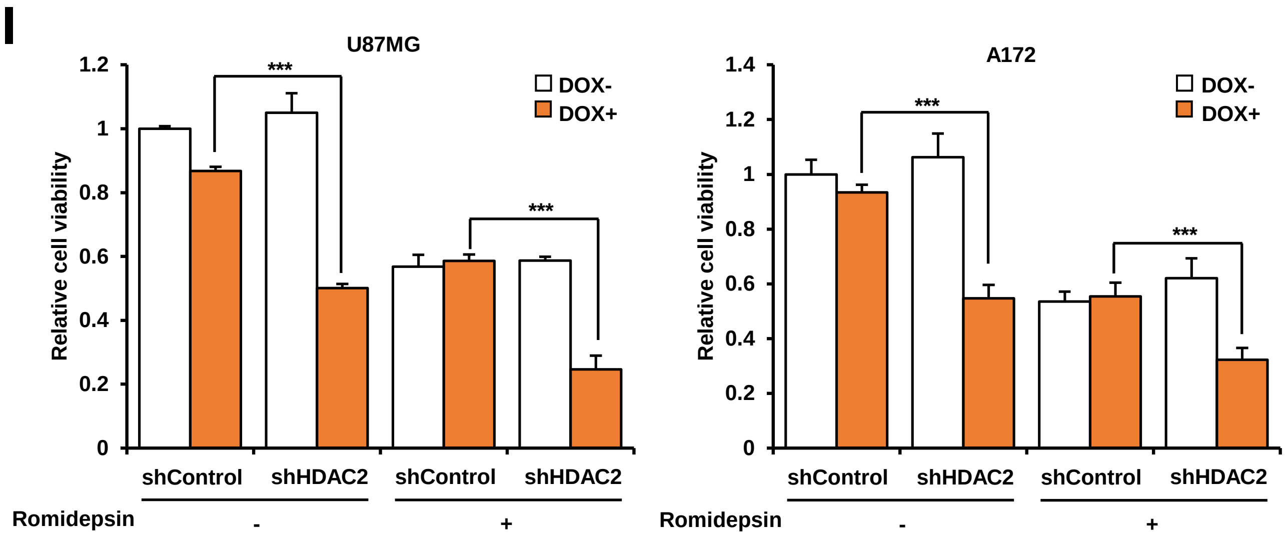

Supplementary Figure 1.

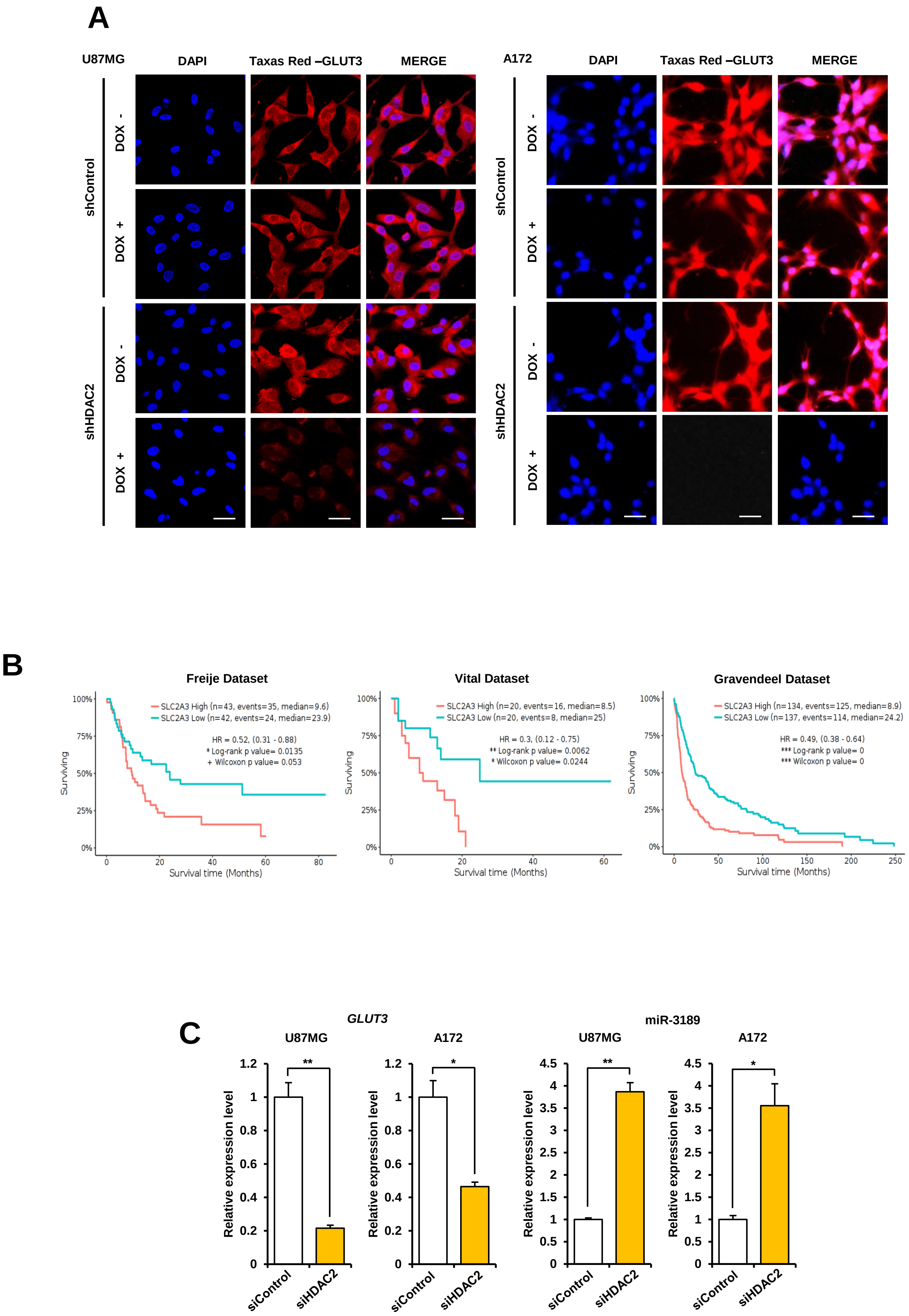

Supplementary Figure 2.

D

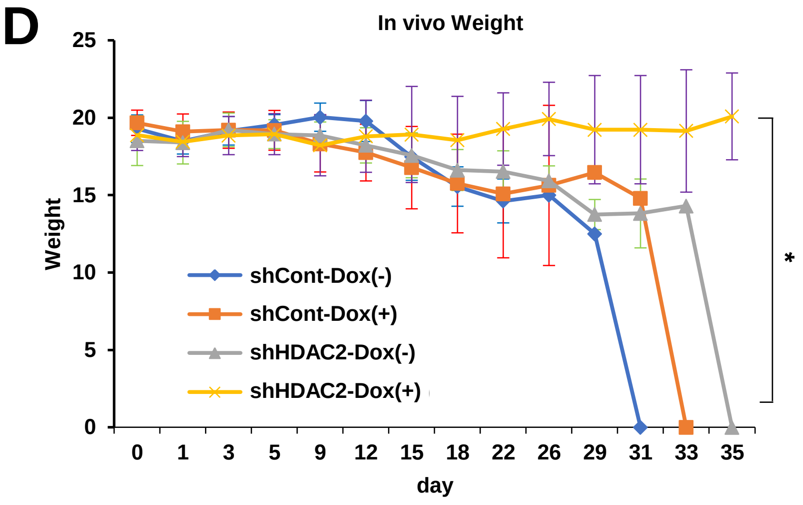

E

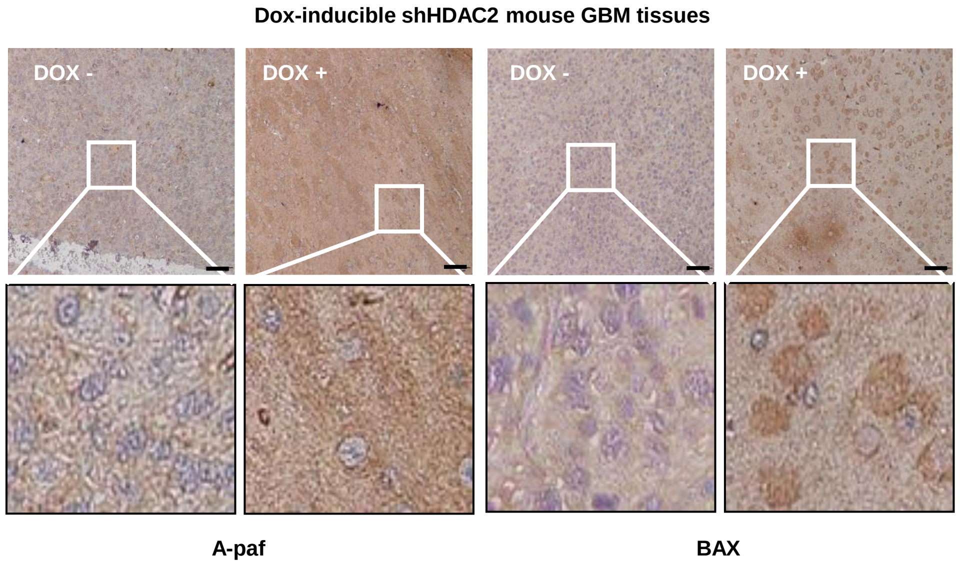

F

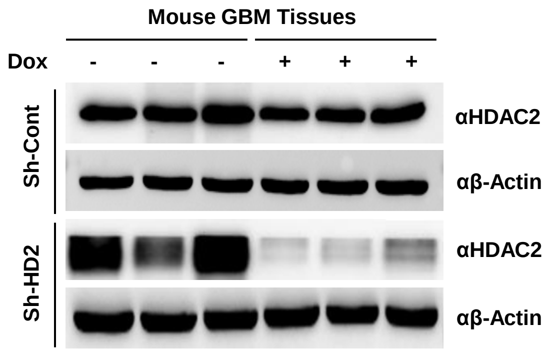

Supplementary Figure 2.

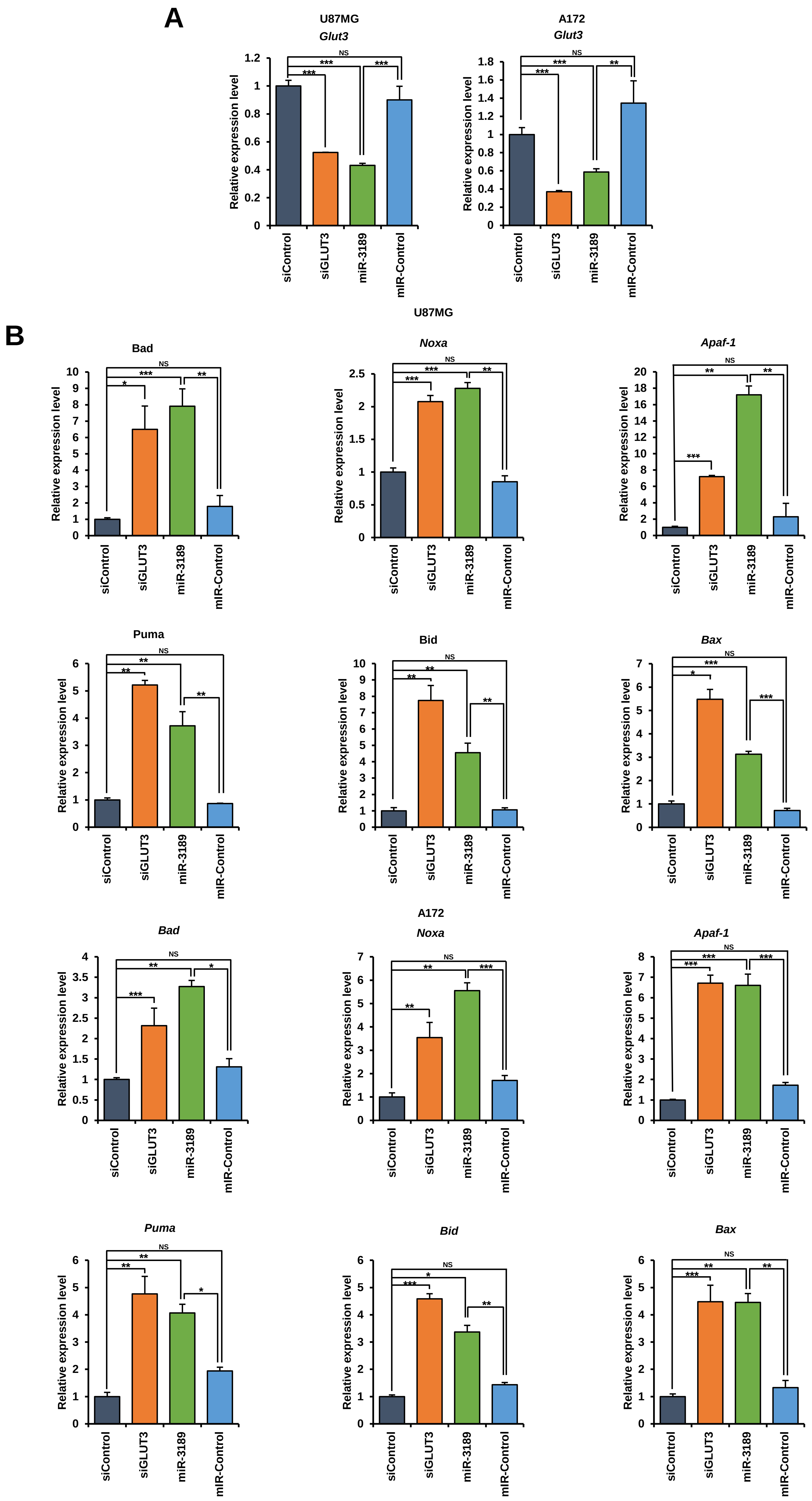

Supplementary Figure 3.

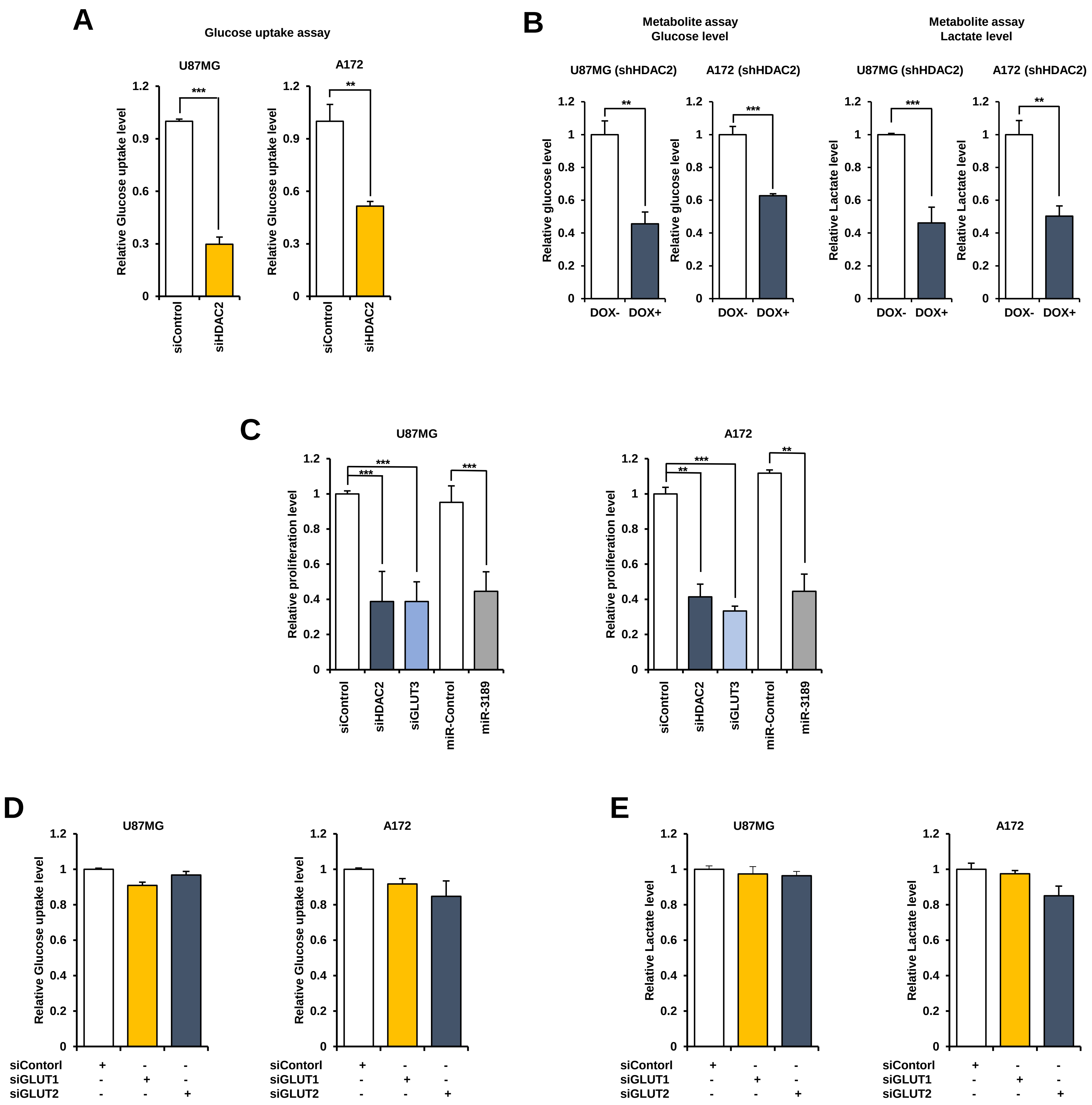

Supplementary Figure 4.

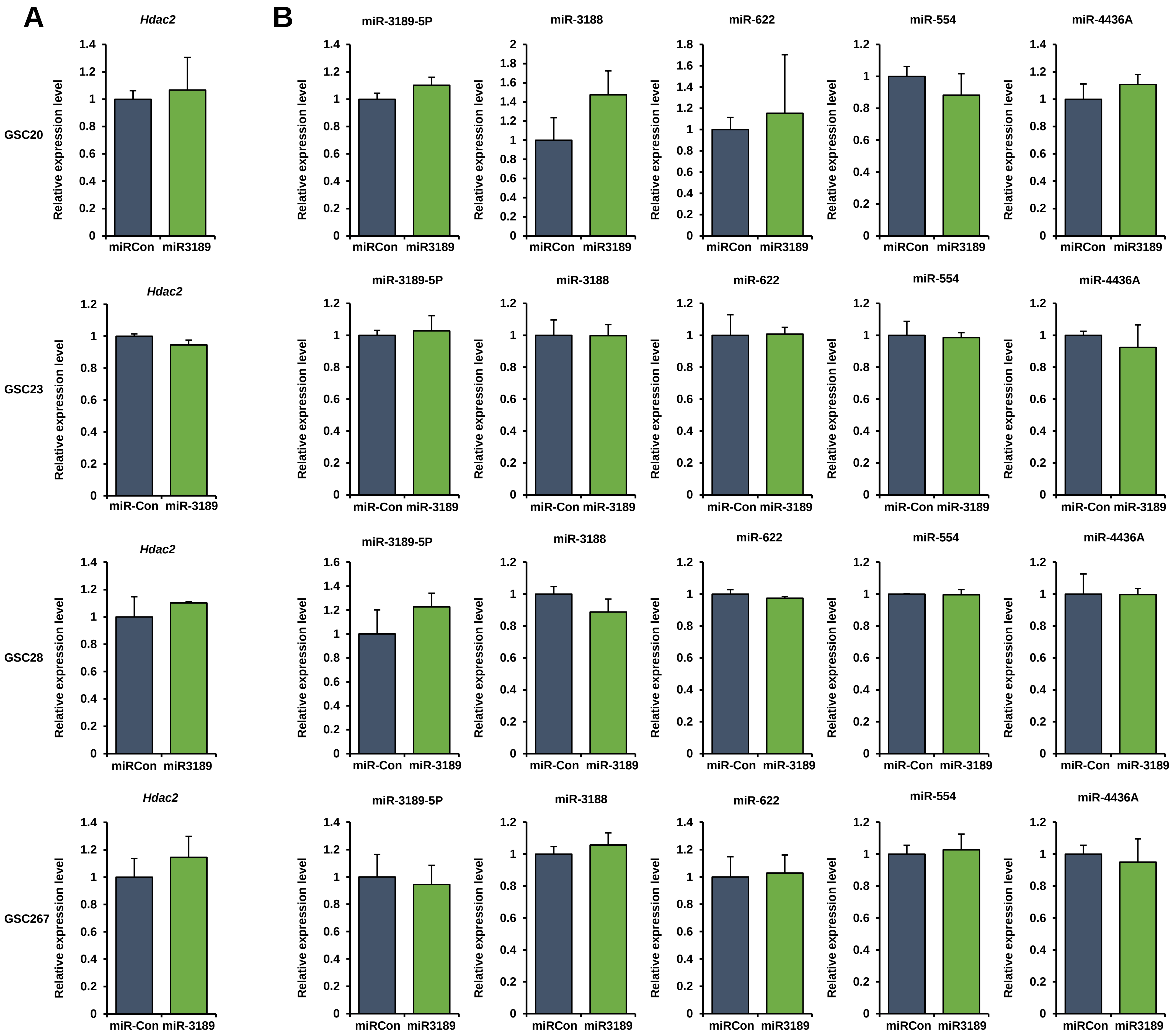

Supplementary Figure 5.
