# Supplementary material for: miR-3189-targeted GLUT3 repression by HDAC2 knockdown inhibits glioblastoma tumorigenesis through regulating glucose metabolism and proliferation
Source: J Exp Clin Cancer Res. 2022 Mar 8;41:87. doi: 10.1186/s13046-022-02305-5 (PMC8903173; doi:10.1186/s13046-022-02305-5)
Supplement: Supplementary file 2 — Additional file 2: Supplementary Table 1. List of primer for qRT-PCR. Supplementary Table 2. Complete list of materials and reagents for experimental. [file 13046_2022_2305_MOESM2_ESM.pdf]

Table 1. Complete list of materials and reagents for experimental

Antibodies		
Anti-caspase 3	Cell signaling	Cat#9662S
HDAC1	Santa cruz	Cat#SC-7872
HDAC2	Santa cruz	Cat#SC-9959
HDAC3	Santa cruz	Cat#SC-376957
HDAC8	Santa cruz	Cat#SC-11405
ACTIN	Sigma	Cat#A5441
PARP	Cell signaling	Cat#9542S
Cleaved Caspase3	Cell signaling	Cat#9661S
BAX	Cell signaling	Cat#5023S
Apaf	Cell signaling	Cat#8969S
P21	Cell signaling	Cat#2947S
GLUT3	Abcam	Cat#ab15311
GFP	Santa cruz	Cat#SC-9996
IgG-Texas Red	Santa cruz	Cat#SC-2780

Biological Samples		
Human Brain Glioma Tissue	US Bio	BS17016b

Chemicals, Peptides, and Recombinant Proteins		
DMEM F12 media	Wellgene	LM002-04
DMEM media	Cellgro	10-013-CV
Dialyzed FBS	Cellgro	35-015-CV
Doxycycline monohydrate	Sigma	D1822
B-27	Gibco	17504-044
DMEM F12 media	Wellgene	LM002-04
DMEM media	Cellgro	10-013-CV
Dialyzed FBS	Cellgro	35-015-CV
Doxycycline monohydrate	Sigma	D1822
B-27	Gibco	17504-044
Romidepsin	Selleckchem	S3020

Critical Commercial Assays		
Agilent Seahorse XF24 Cell Culture Microplate	Agilent Technologies	100777-004
Agilent Seahorse XF24 Extracellular Flux Assay Kit	Agilent Technologies	103020-100
2-DG uptake measurement kit(25tests)	Cosmobio	CSR-OKP-PMG-K01TE
Lactate Colorimetric/Flurometiric Assay Kit	Biovision	K607
Mouse and Rabbit Specific HRP/DAB (ABC) Detection IHC kit	Abcam	ab64264
Total RNA lsolation KIT	Intron	17221
TUNEL Assay kit - In situ Direct DNA	Abcam	ab66108

Experimental Models: Cell Lines

Human: U87MG glioblastoma cell line, male	ATCC	CRL-HTB14
Human: SVG P-12	ATCC	CRL-8621
Human: A172 glioblastoma cell line, male	ATCC	CRL-1620
Human: T98G glioblastoma cell line, male	ATCC	CRL-1690
Human: LN229 glioblastoma cell line, male	ATCC	CRL-2611
Human: U118 glioblastoma cell line, male	ATCC	CRL-HTB15
Human: U343 glioblastoma cell line, male	ATCC	CRL-701163
Human: U373 glioblastoma cell line, male	ATCC	CRL-HTB17
Human: Glioma stem cell – GSC 267	E.P. Sulman, M.D., Ph.D.	N/A
Human: Glioma stem cell – GSC 20	E.P. Sulman, M.D., Ph.D.	N/A
Human: Glioma stem cell – GSC 23	E.P. Sulman, M.D., Ph.D.	N/A
Human: Glioma stem cell – GSC 28	E.P. Sulman, M.D., Ph.D.	N/A

**Experimental Models: Organisms/Strains**		
BALB/C$^{nu/nu}$ mice	Central Lab Animal	SLC-M-0386

**Recombinant DNA**		
Tet-pLKO-puro (TetOn)	addgene	21915
pGL3-Basic vector	addgene	E1751
pmirGLO Dual-Luciferase miRNA Target Expression Vector	Promega	E1330
pLKO-shRNA HDAC1 #1	Sigma	TRCN0000004816
pLKO-shRNA HDAC1 #2	Sigma	TRCN0000004818
pLKO-shRNA HDAC2 #1	Sigma	TRCN0000196590
pLKO-shRNA HDAC2 #2	Sigma	TRCN0000004821
pLKO-shRNA HDAC3 #1	Sigma	TRCN0000195333
pLKO-shRNA HDAC3 #2	Sigma	TRCN0000004825
pLKO-shRNA HDAC8 #1	Sigma	TRCN0000004850
pLKO-shRNA HDAC8 #2	Sigma	TRCN0000004851

**siRNA and miRNA**		
siRNA Control	Bioneer	#SN-1001
siRNA HDAC8	Bioneer	#1067643
siRNA HDAC1	Bioneer	#1067571
siRNA HDAC2	Bioneer	#1067581
siRNA HDAC3	Bioneer	#1067595
siRNA GLUT3	Bioneer	#1139312
miRNA Negative Control, mimic	Bioneer	#SMC-2001
miRNA 3189, mimics	Bioneer	#SMM-002
Tet-pLKO-puro (TetOn) shRNA Control	This paper	N/A
Tet-pLKO-puro (TetOn) shRNA HDAC2	This paper	N/A
pmirGLO GLUT3 WT binding site – (miR-3189 target)	This paper	N/A

pmirGLO GLUT3 MT binding site – (miR-3189 target)	This paper	N/A
pGL3-Basic Control reporter	This paper	N/A
pGL3-Basic Puma reporter	This paper	N/A

**Software and Algorithms**		
ImageJ	Https://doi.org/10.1038/nmeth.2089	https://imagej.nih.gov/ij/
ZEN blue	Zeiss	http://www.zeiss.com/micorscopy/int/products/microscope-software/zen-lite.html
Graphpad Prism	Graphpad software	N/A
GELCOUNT	Oxford Optronix	https://www.oxford-optronix.com/gelcount
Primer Express 3.0.1	https://www.thermofisher.com/order/catalog/product/4363991#/4363991	4363991

Table 2. List of primer for qRT-PCR

Primer sequences		
**HDAC1**<br>Fwd CAA AGG ACA CGC CAA GTG TG<br>Rev AGG AAT CGC CTG CAT TTG GA	Macrogen	N/A
**HDAC2**<br>Fwd ATG GCG TAC AGT CAA GGA GG<br>Rev ACT GAA CCG CCA GTT GAG AG	Macrogen	N/A
**HDAC3**<br>Fwd TGT GAT CGA TTG GGC TGC TT<br>Rev TGC AGG CAC GTC ATG AAT CT	Macrogen	N/A
**HDAC8**<br>Fwd TGA CTC CAG TGG GAA TTG GC<br>Rev ACC ACA TGC TTC AGA TTC CCT	Macrogen	N/A
**GLUT3**<br>Fwd AAC CAG CTG GGC ATC GTT GTT GG<br>Rev GCC ACA ATA AAC CAG GGA ATG GG	Macrogen	N/A
**GAPDH**<br>Fwd TGATGACATCAAGAAGGTGGTGAAG<br>Rev TCCTTGGAGGCCATGTAGGCCAT	Macrogen	N/A
**BAX**<br>Fwd GGT TTC ATC CAG GAT CGA GAC GG<br>Rev ACA AAG ATG GTC ACG GTC TGC C	Macrogen	N/A
**BID**<br>Fwd CCA CAC CGT GGT CTT TCC AGC<br>Rev GGC TGG AAC CGT TGT TGA CC	Macrogen	N/A
**BAD**<br>Fwd ACT GAG GTC CTG AGC CGA CA<br>Rev CGG CTC AAA CTC TGG GAT CTG	Macrogen	N/A
**APAF**<br>Fwd TCC CGG GCA AAA GGG ATA GA<br>Rev CCC CAC TAC TGG ACA CAA AGG	Macrogen	N/A
**PUMA**<br>Fwd CAA ACC AGA GCA GGG CAG GAA G<br>Rev GCT CCC TGG GGC CAC AAA TCT	Macrogen	N/A
**P53**<br>Fwd GAA CAA GTT GGC CTG CAC TG<br>Rev GAA GTG GGC CCC TAC CTA GA	Macrogen	N/A
**GLUT1**<br>Fwd AAG TCC TTT GAG ATG CTG ATC CT<br>Rev AAG ATG GCC ACG ATG CTC AGA TA	Macrogen	N/A
**P21**<br>Fwd GTC ACT GTC TTG TAC CCT TGT G<br>Rev CGG CGT TTG GAG TGG TAG AAA	Macrogen	N/A
**HTRA2**<br>Fwd ATC GCA ACG CTG AGG ATT CA<br>Rev CCC ATC CTG CAG GGA TAC AG	Macrogen	N/A
**Survivin**<br>Fwd TGG CCG CTC CTC CCT CAG AAA A<br>Rev GCT GCT GCC TCC AAA GAA AGC G	Macrogen	N/A
**XIAP**<br>Fwd CAC TTG AGG TTC TGG TTG CAG<br>Rev TGC AAA GCT TCT CCT CTT GC	Macrogen	N/A
**HSP60**<br>Fwd ATT CCA GCA ATG ACC ATT GC<br>Rev GAG TTA GAA CAT GCC ACC TC	Macrogen	N/A

**Poly adaptor**<br>GCG AGC ACA GAA TTA ATA CGA CTC ACT AT AGGT TTT TTT TTT	Macrogen	N/A
**Poly adaptor reverse**<br>GCG AGC ACA GAA TTA ATA CGA C	Macrogen	N/A
**miR622**<br>Fwd ACA GTC TGC TGA GGT TGG AGC	Macrogen	N/A
**miR3188**<br>Fwd AGA GGC TTT GTG CGG ATA CGG GG	Macrogen	N/A
**miR4436**<br>Fwd GCA GGA CAG GCA GAA GTG GAT	Macrogen	N/A
**miR544B**<br>Fwd ACC TGA GGT TGT GCA TTT	Macrogen	N/A
**miR3189**<br>Fwd CCC TTG GGT CTG ATG GGG TAG	Macrogen	N/A
**miR924**<br>Fwd AGA GTC TTG TGA TGT CTT GC	Macrogen	N/A
**miR581**<br>Fwd TGT GTG ATC TAA AGA ACA	Macrogen	N/A
**miR4677**<br>Fwd GAC CTT CTG TCT GTG AGA	Macrogen	N/A
**miR3939**<br>Fwd TAC GCG CAG ACC ACA GGA TGT C	Macrogen	N/A
**miR421**<br>Fwd ATC AAC AGA CAT TAA TTG GGC GC	Macrogen	N/A
**miR4324**<br>Fwd CCC TGA GAC CCT AAC CTT AA	Macrogen	N/A
**miR137**<br>Fwd ACG GGT ATT CTT GGG TGG	Macrogen	N/A
**miR516b**<br>Fwd ATC TGG AGG TAA GAA GCA CTT T	Macrogen	N/A
**miR548H3**<br>Fwd AAA AGT AAT CGC GGT TTT	Macrogen	N/A
